# Oleanolic Acid Ameliorates Metabolic Dysfunction-Associated Steatotic Liver Disease by Inhibiting Ferroptosis through Targeting PTGS2 as a Key Molecular Node and Activating the AMPK/ACC Signaling Pathway

**DOI:** 10.1021/acs.jafc.5c17633

**Published:** 2026-07-01

**Authors:** Wei Ye, Lei Wang, Ye Fan, Li Ma, Zaohong Chen, Jinmeng Zhang, Rong Zeng, Xiang Liu

**Affiliations:** † Department of Laboratory Medicine, 240515 Hubei University of Chinese Medicine, Wuhan, Hubei 430065, China; ‡ Hubei Shizhen Laboratory, Hubei University of Chinese Medicine, Wuhan, Hubei 430065, China; § Department of Chinese Medicine, 240515 Hubei University of Chinese Medicine, Wuhan, Hubei 430065, China; ∥ Department of Spleen and Gastroenterology, Hubei Provincial Hospital of Traditional Chinese Medicine, Hubei University of Chinese Medicine, Wuhan, Hubei 430061, China

**Keywords:** MASLD, ferroptosis, oleanolic acid, network pharmacology, AMPK/ACC, PTGS2

## Abstract

Metabolic dysfunction-associated steatotic liver disease (MASLD) progression closely involves ferroptosis. Using high-fat diet-challenged mice and free fatty acid (FFA)-treated HepG2 cells, we demonstrate that oleanolic acid (OA) ameliorates MASLD and suppresses ferroptosis—an effect validated by the ferroptosis activator Erastin. Supported by network pharmacology, mechanistic analyses reveal that OA exerts synergistic efficacy via a dual-axis network. First, molecular docking, cellular thermal shift assay (CETSA), and molecular dynamics (MD) simulations confirm that OA directly binds PTGS2, mitigating lipid peroxidation and inflammatory mediator release. Second, OA activates the AMPK/ACC metabolic signaling pathway, inhibiting ACC through phosphorylation to correct lipid metabolism disorders, and its antiferroptotic effect can be blocked by AMPK inhibitors. This study first elucidates OA’s therapeutic mechanism against MASLD via “metabolic correction + oxidative inhibition,” outlining a novel strategy for targeting hepatic ferroptosis.

## Graphical abstract

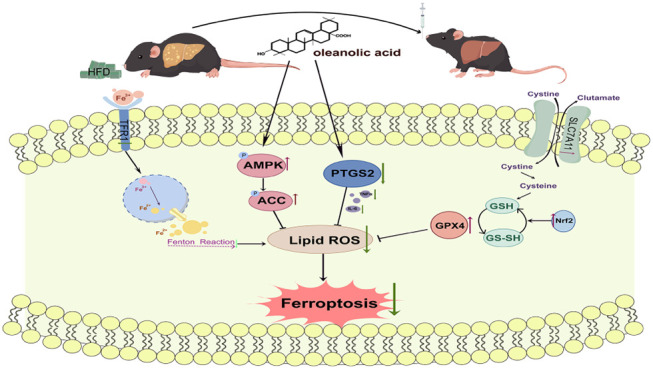

## 1 Introduction

Metabolic dysfunction-associated steatotic liver disease (MASLD), the revised designation of former nonalcoholic fatty liver disease (NAFLD), constitutes the predominant chronic liver disease worldwide, with nearly 30% of global residents suffering from this illness and its morbidity continuing to rise.^1–3^ Its progression can advance from NAFLD to nonalcoholic steatohepatitis (NASH), cirrhosis, and even hepatocellular carcinoma (HCC),^4^ posing a serious threat to human health. Currently, there are no approved targeted therapeutic drugs available clinically, with management primarily based on routine lifestyle modification covering nutritional optimization and enhanced daily activity to reduce weight. However, these approaches often face poor compliance and yield limited efficacy. Therefore, elucidating novel mechanisms underlying MASLD progression and identifying effective intervention strategies are critically important.

Ferroptosis—an iron-based type of regulatory cell death—has been proven to be a pivotal inducer for MASLD/MASH progression.^5^ The hallmark of ferroptosis is excessive intracellular iron accumulation accompanied by uncontrolled lipid peroxidation. Its core mechanism involves impaired function of the light chain subunit *SLC7A11* belonging to cystine/glutamate reverse transporter System Xc-, which results in suppressed biosynthesis and consumption of the intracellular antioxidant glutathione (GSH). GSH depletion reduces or inactivates the crucial antioxidant enzyme glutathione peroxidase 4 (GPX4), preventing clearance of toxic lipid peroxides. This triggers catastrophic buildup of reactive oxygen species (ROS) and generation of lipid radicals. Furthermore, disrupted hepatic iron homeostasis exacerbates this oxidative damage.^6^ As ferroptosis directly drives hepatocyte death and inflammatory responses, it presents a highly promising novel therapeutic target for intervening in MASLD/MASH progression, making it a current hotspot in liver disease research.

In the pathophysiology of ferroptosis, prostaglandin synthase 2 (*PTGS2*, namely *COX-2*) expression is often used as a key biomarker for lipid peroxidation and ferroptosis occurrence.^7,8^ *PTGS2* metabolizes intracellularly accumulated polyunsaturated fatty acids (PUFAs) and participates in the production of lipid mediators such as prostaglandins. More importantly, PTGS2 serves as the rate-controlling enzyme in the inflammatory response, and its upregulation promotes the synthesis of inflammatory mediators.^9^ This exacerbates inflammatory injury and fibrosis in hepatocytes, directly driving progression from MASLD to MASH. Consequently, *PTGS2* not only catalyzes the production of pro-inflammatory lipid mediators to intensify hepatic inflammation but is also closely involved in the metabolism of lipid peroxidation substrates required for ferroptosis. This suggests it may serve as a pivotal node linking inflammation and ferroptosis.

On the other hand, energy metabolism disorders represent another core feature of MASLD. The AMPK signaling pathway, acting as a cellular energy sensor, is typically recognized for its negative regulation of ferroptosis.^10^ Mechanistically, activated AMPK inhibits de novo lipogenesis via phosphorylating and suppressing the activity of acetyl-CoA carboxylase (ACC), thereby reducing de novo lipogenesis. This regulatory mechanism effectively reduces intracellular pools of polyunsaturated fatty acids (PUFAs), substrates prone to lipid peroxidation.^11,12^ Thus, at the metabolic source, it jointly determines hepatic cell susceptibility to ferroptosis alongside PTGS2-mediated oxidative processes. Consequently, the AMPK/ACC signaling axis serves as a pivotal regulatory node linking lipid metabolism and ferroptosis.

Given the lack of effective drugs for MASLD, identifying natural, low-toxicity compounds from traditional Chinese medicine holds significant research value. Oleanolic acid (OA), a naturally derived pentacyclic triterpenoid abundantly distributed within edible vegetation (e.g., olives, hawthorn), exhibits potent anti-inflammatory, antioxidant, antitumor, and hypolipidemic activities.^13,14^ Of particular note, as a natural antioxidant, OA alleviates oxidative stress by effectively neutralizing ROS, a primary pathological driver of numerous metabolic disorders.^15^ Extensive research confirms that oleanolic acid demonstrates promising efficacy and significant application potential in treating metabolic syndrome.^16–19^ However, its potential remains largely unexplored in the field of MASLD. Although preliminary studies suggest OA may improve MASLD, relevant literature remains scarce. Existing mechanistic explorations primarily focus on lipid metabolism regulation,^20,21^ with insufficient investigation into other potential pathways. Whether its efficacy in MASLD involves ferroptosis regulation and its specific molecular mechanisms—particularly whether it simultaneously targets PTGS2 and AMPK/ACC through a potential dual-axis synergistic mechanism—remains unexplored.

Against this backdrop, we propose the scientific hypothesis that OA may exert its therapeutic effect on MASLD by simultaneously inhibiting PTGS2-mediated lipid peroxidation-induced inflammatory responses and activating the AMPK/ACC pathway. This dual-axis synergistic inhibition of hepatic ferroptosis thereby improves lipid metabolic homeostasis. This study aims to validate the therapeutic efficacy of OA using in vivo plus in vitro MASLD models and to elucidate its specific molecular mechanisms for inhibiting ferroptosis via

the PTGS2-AMPK/ACC axis, thereby providing new theoretical foundations and candidate strategies for treating MASLD.

## 2 Materials and Methods

### 2.1 Animal Experiments

All animal procedures strictly adhered to established regulations and guidelines governing laboratory animal welfare. Experimental protocols obtained authorization from the Animal Welfare Ethics Committee of Wuhan Luobin Life Technology Co., Ltd. (Approval No.: LBSM2025023). SPF-grade male C57BL/6J mice (6 weeks old) were bought from Skobes Biotech, Henan, China. All mice were raised inside a standard laboratory under ambient temperature of (22 ± 2) °C, 45%–55% relative humidity and a 12 h light-dark cycle, noise levels below 55 dB, and provided with food and water ad libitum.

Following a seven-day acclimatization period, the murine subjects were allocated via randomization into five distinct groups: (1) Control (CON) group (n = 6), (2) High-Fat Diet (HFD) group (n = 6), (3) HFD plus low-dose oleanolic acid (HFD + OA-L, 60 mg/kg) group (n = 6), (4) HFD plus high-dose oleanolic acid (HFD + OA-H, 120 mg/kg) group (n = 6), and (5) HFD plus positive control fenofibrate (HFD + Feno, 40 mg/kg) group (n = 6).

OA (Purity ≥98%, O8260, Solarbio, Beijing, China) was dissolved in PBS (pH 7.2–7.4) supplemented with 2% Tween 80. Fenofibrate (Purity ≥98%, Aladdin, Shanghai, China). Except for the control group mice fed a normal chow diet (NCD), all other groups were fed a high-fat diet (HFD; 60% fat, 20% protein, 20% carbohydrate, catalog no.: HF60, Dyets Biotechnology (Wuxi) Limited). Comprehensive dietary formulations are delineated in Table S1. After 10 weeks of feeding,^22^ HFD + OA-L, HFD + OA-H, and HFD + Feno groups received oral administration via gavage, while the CON and HFD groups received an equal volume of 2% Tween 80 phosphate-buffered saline. Treatment continued for 8 weeks. Dosing and timing were determined by reference to studies.^21,23,24^

### 2.2 Histological Staining

Fresh hepatic specimens were fixed in fixative (Catalog No.: G1101, Servicebio, Wuhan, China.). After paraffin embedding, the tissues were sectioned into 4-μm sections prior to conventional H&E staining. For lipid detection, samples were frozen-embedded under cold conditions, cut into 10-μm sections, stained with Oil Red O to visualize lipids, and counterstained with hematoxylin to visualize nuclei. Images of H&E-stained and Oil Red O-stained sections were captured using an Olympus microscope. For immunohistochemical (IHC) staining, 4-μm sections were deparaffinized in xylene, rehydrated, and then treated with microwave antigen retrieval (0.01 M citrate buffer, pH 6.0). Endogenous peroxidase was inhibited with 3% H_2_O_2_, followed by overnight incubation at 4 °C with GPX4 primary antibody (1:4000, clone 3F5G5, 67763-1-Ig, Proteintech Group, Inc.), followed by incubation with biotinylated secondary antibody. Staining was performed using DAB chromogen solution, with nuclei counterstained using hematoxylin. Finally, after dehydration, clearing, and mounting, GPX4 expression was observed under a microscope.

### 2.3 Serum Assays

Multiple serum parameters were assessed, including total cholesterol (TC, A111–1–1), triglycerides (TG, A110–1–1), low-density lipoprotein cholesterol (LDL-C, A113–1–1), high-density lipoprotein cholesterol (HDL-C, A112–1–1), alanine aminotransferase (ALT, C009–2–1), and aspartate aminotransferase (AST, C010–2–1). All assays were conducted with reagents from Nanjing Jiancheng, China. Furthermore, circulating levels of tumor necrosis factor (TNF-α, EK282EG) and interleukin-6 (IL-6, EK206EGA) were quantified via ELISA kits (MultiSciences, Hangzhou, China).

### 2.4 Determination of MDA, SOD, ROS, GSH, and Fe^2+^ Levels in Liver Tissue

Tissue samples were homogenized on ice in physiological saline or the kit-specified buffer at a 1:9 weight/volume ratio. The resulting homogenates were centrifuged at 12,000 × g for 10 min at 4 °C, and the supernatants were collected for subsequent analysis. The BCA Protein Concentration Assay Kit (E-BC-K318-M, Elabscience, Wuhan, China) was adopted to quantify total protein concentration. Levels of malondialdehyde (MDA), superoxide dismutase (SOD) activity, ROS (detected by DHE fluorescence), GSH and ferrous ion (Fe^2+^) were determined strictly following the manufacturers’ instructions. The assay kits used were as follows: A003–1 and A001–3 (Nanjing Jiancheng), G1746–100T (Servicebio), BC1175 (Solarbio), and E-BC-K773-M (Elabscience).

### 2.5 Cell Culture and Treatment

HepG2 hepatocellular carcinoma cells were procured from SUNNCELL (catalog no. SNL-083; sunncell.com.cn). Short tandem repeat (STR) profiling verified that the genetic identity of this line corresponds to the HepG2 reference strain (ACC-180) archived in the DSMZ database. Cellular propagation was conducted within a humidified environment at 37 °C under 5% CO_2_ tension, utilizing high-glucose DMEM fortified with 10% fetal bovine serum (FBS) and 1% penicillin-streptomycin antibiotic solution.

Based on our previous studies^25^ and findings from others,^26^ FFA was selected to induce intracellular lipid accumulation and ferroptosis. The FFA solution comprised oleic acid (OLA): palmitic acid (PA) = 2:1 (OLA: 400 μM, PA: 200 μM). Cells were cultured to 80% confluence, then incubated in serum-free DMEM for 6 h before treatment with DMEM containing OA (0–125 μM) and FFA solution for 24 h.

When using the AMPK inhibitor Compound C (GC17243, GLPBIO), after reaching 80% confluence and incubating in serum-free DMEM for 6 h, add DMEM containing 10 μM Compound C for 2 h, followed by treatment with DMEM containing OA and FFA solution for 24 h. When using the ferroptosis inducer Erastin (HY-15763, MedChemExpress), cells were cultured to 60% confluence and rested in serum-free DMEM for 6 h before adding DMEM containing 10 μM Erastin for 24 h. Cells were then exposed to OA-containing DMEM for an additional 24 h. For treatment with the PTGS2 activator 6-OHDA (MB5924–1, Meilun, Dalian, China), cells at 60% confluence were first synchronized via serum starvation in DMEM for 6 h. Then, the cellular cultures underwent a 24-h incubation period with 100 μM 6-OHDA, followed by a subsequent 24 h treatment with DMEM containing OA and FFA.

### 2.6 Cell Viability Assay

Cells were plated in 96-well formats and cultured until reaching approximately 80% confluency, followed by exposure to oleanolic acid at concentrations of 0, 5, 10, 25, 50, 75, 100, or 125 μM for 24 h. Subsequent evaluation of cellular viability was conducted utilizing the CCK-8 proliferation assay kit (CK04; Dojindo, Beijing, China). Following a 1 h incubation with the reagent at 37 °C, optical density readings were acquired at 450 nm via an Epoch 2 microplate spectrophotometer (BioTek, USA) to quantify viable cell numbers.

### 2.7 Cell Oil Red O Staining

Following two rinses with PBS, the cells underwent fixation for 25 min. Subsequent to another pair of PBS washes, a 1 min treatment with 60% isopropanol was applied to eliminate residual fixative, thereby optimizing conditions for Oil Red O (O8010, Solarbio, Beijing, China) staining. Subsequently, Oil Red O working solution (stock: ddH_2_O = 3:2) was applied to cells for 20 min of staining. Following a rinse in 60% isopropanol, nuclear counterstaining was completed with hematoxylin (BL700B, Labgic) for 2 min. Finally, the stained images were analyzed using ImageJ software.

### 2.8 Evaluation of Cellular Lipid Deposition, Lipid Peroxidation, and Antioxidant Levels

Cells received dual PBS washes ahead of testing, followed by 30 min of lysis on ice with RIPA buffer (E-BC-R327, Elabscience, Wuhan, China) containing 1% PMSF and 1% Na_3_VO_4_. The levels of TC, TG, MDA, SOD, and GSH were measured using the corresponding assay kits as described for the animal experiments. All data were normalized to total protein.

### 2.9 Intracellular ROS and Fe^2+^ Level Assay

Following two rounds of rinsing using serum-deprived DMEM, the reactive oxygen species (ROS) detection kit (MA0219, Meilun, Dalian, China) was used. Cells were incubated with DMEM containing 10 μM DCFH-DA for 30 min at 37 °C under light-protected conditions. Subsequently, the samples were rinsed thrice with serum-free DMEM. Fluorescent images were captured and processed via Nikon ECLIPSE 80i fluorescence microscope. Cellular ferrous ion concentrations were quantified with the Cellular Ferrous Ion Assay Kit (E-BC-K881-M, Elabscience, Wuhan, China), and cells were counted. Results were normalized to cell number.

### 2.10 Network Pharmacology Analysis

Target genes associated with MASLD were screened out from GeneCards with “non-alcoholic fatty liver disease” set as a retrieval keyword.^27^ PubChem database was adopted to acquire the SMILES string of oleanolic acid.^28^ This was further uploaded to the SwissTargetPrediction database to identify targets of OA (probability >0).^29^ A Venn graph was plotted on the online bioinformatics Web site to filter shared genes between OA targets and MASLD-related genes. These genes were analyzed via the STRING database^30^ to build a protein–protein interaction (PPI) network. Subsequently, the generated PPI network was graphically rendered with Cytoscape 3.10.0 software.^31^ Within Cytoscape, these intersecting genes were scored to obtain degree values, and genes with degrees >median were selected as core genes. These core genes were uploaded to the DAVID database for GO and KEGG pathway enrichment analysis.^32^ Pathways with *p* < 0.05 were considered significantly enriched. Visualization of results was performed using the MicroBioinformatics Web site.

### 2.11 Molecular Docking

The 2D structures of small-molecule ligands retrieved from the PubChem database were imported into ChemOffice to generate corresponding 3D structures. Receptor proteins were screened from the RCSB PDB database and pretreated using PyMOL 2.6 to remove water molecules and phosphate groups. AutoDock 1.5.6 was used to add polar hydrogens to macromolecules, set rotatable bonds for ligands, and define the grid box coordinates. Subsequently, molecular docking was performed via AutoDock Vina, and the optimal protein–ligand binding conformation was selected according to docking scores. Finally, Discovery Studio 2019 and PyMOL 2.6 were applied for result visualization. All database URLs are provided in Table S2.

### 2.12 Molecular Dynamics Simulation

Molecular dynamics simulations (100 ns) were run in GROMACS 2022. The receptor adopted the AMBER14SB force field, and the ligand’s RESP-charged GAFF2 topology was built via sobtop_1.0 (dev3.1). The system was solvated with TIP3P water (1 nm box buffer) and neutralized by 0.15 mol/L NaCl to simulate physiological environments.^33^ Long-range electrostatics were calculated by PME (1 nm cutoff), with bond constraints handled by the LINCS algorithm. After three-step energy minimization (3000 steepest descent +2000 conjugate gradient cycles), 100 ns NPT production simulations were performed with a 2 fs time step. The Nosé-Hoover thermostat and Parrinello–Rahman barostat stabilized the system at 310 K and 1 bar. Trajectory analyses included root-mean-square deviation (RMSD), root-mean-square fluctuation (RMSF), hydrogen bonds (HBonds), radius of gyration (Rg), solvent-accessible surface area (SASA), and Gibbs free energy to systematically evaluate conformational stability and solvent interactions.

### 2.13 Cell Thermal Shift Assay

Cells were disrupted via three iterative freeze–thaw cycles utilizing liquid nitrogen and a water bath. Following centrifugation, 90 μL aliquots of the supernatant were supplemented with oleanolic acid to attain a final concentration of 50 μM. Conversely, control samples were incubated with DMSO, the vehicle for oleanolic acid at 37 °C for 2 h. Subsequently, the mixtures were transferred into PCR tubes and subjected to thermal denaturation across a gradient of 40, 45, 50, 55, 60, and 65 °C for 3 min using a thermal cycler. After clarification by centrifugation at 15,000 × g for 40 min at 4 °C, the recovered supernatants were combined with loading buffer and boiled at 95 °C for 5 min. The prepared lysates were ultimately analyzed via Western blotting.

### 2.14 Semi-Quantitative PCR (SQ-PCR) Analysis

HepG2 cells were lysed using Trizol reagent (B610409, Sangon, Shanghai, China) to extract total RNA. Subsequently, the RNA was reverse-transcribed into cDNA using a commercial kit (D7170M, Beyotime, Shanghai, China). PCR amplification was performed using the synthesized cDNA as a template, with primer sequences detailed in Table S3. The amplicons were resolved by 1.5% agarose gel electrophoresis and visualized using a Tanon 1600 imaging system. Band optical densities (OD) were analyzed via ImageJ software.

### 2.15 Western Blot Analysis

Cells received dual PBS washes ahead of testing, followed by 30 min of lysis on ice with RIPA buffer containing 1% PMSF and 1% Na_3_VO_4_; cells were scraped and repeatedly pipetted until solution was clear. After centrifugation at 12,000 rpm and 4 °C lasting 10 min, the supernatant was harvested. The samples were quantified via commercial BCA protein quantification kits. Protein samples were diluted with physiological saline to adjust concentration. The prepared samples were mixed with 5× SDS loading buffer and denatured at 95 °C for 10 min. Subsequent protein separation was implemented via 10% SDS-PAGE, and samples were transferred to a PVDF membrane, and blocked at room temperature for 1.5 h with 5% skim milk powder. After three rounds of rinsing with TBST, the blots were incubated overnight at 4 °C with corresponding primary antibodies. After three washes with TBST, membranes were incubated with an HRP-conjugated goat antirabbit secondary antibody for 40 min. Finally, bands were visualized with ECL Ultra-Sensitive Chemiluminescent Reagent Kit, followed by image acquisition via a chemiluminescence imaging device. ImageJ quantification: target protein band density normalized to internal control. All antibodies and reagents for Western blot are provided in Table S4.

### 2.16 Statistical Analysis

Statistical analysis was performed using GraphPad Prism 10.1.2. Results are expressed as mean ± standard deviation (SD) of at least three independent biological replicates. Data sets were assessed for normality and variance homogeneity. When these assumptions were met, one-way analysis of variance (ANOVA) was used for comparisons among three or more groups; otherwise, appropriate nonparametric tests were applied. Statistical significance was defined as * *p* < 0.05, ** *p* < 0.01, or *** *p* < 0.001.

## 3 Results

### 3.1 OA Modulates Intracellular Lipid Deposition

First, we used the CCK-8 assay to assess the impact of different OA concentrations on cell viability. We found that OA concentrations between 0 and 50 μM showed no significant influence on HepG2 cell viability (Figure 1A). Next, we established a cellular model in HepG2 cells using FFA solution. Oil Red O staining showed OA reduced lipid droplets induced by the FFA solution, with the most evident effect observed at 50 μM (Figure 1B–C). Similarly, OA decreased TC and TG levels following FFA solution treatment, exhibiting a dose-dependent reduction trend with the strongest effect at 50 μM (Figure 1D–E). Based on these findings, we confirmed 50 μM as the optimal intervention concentration for subsequent studies.

**Figure 1:**
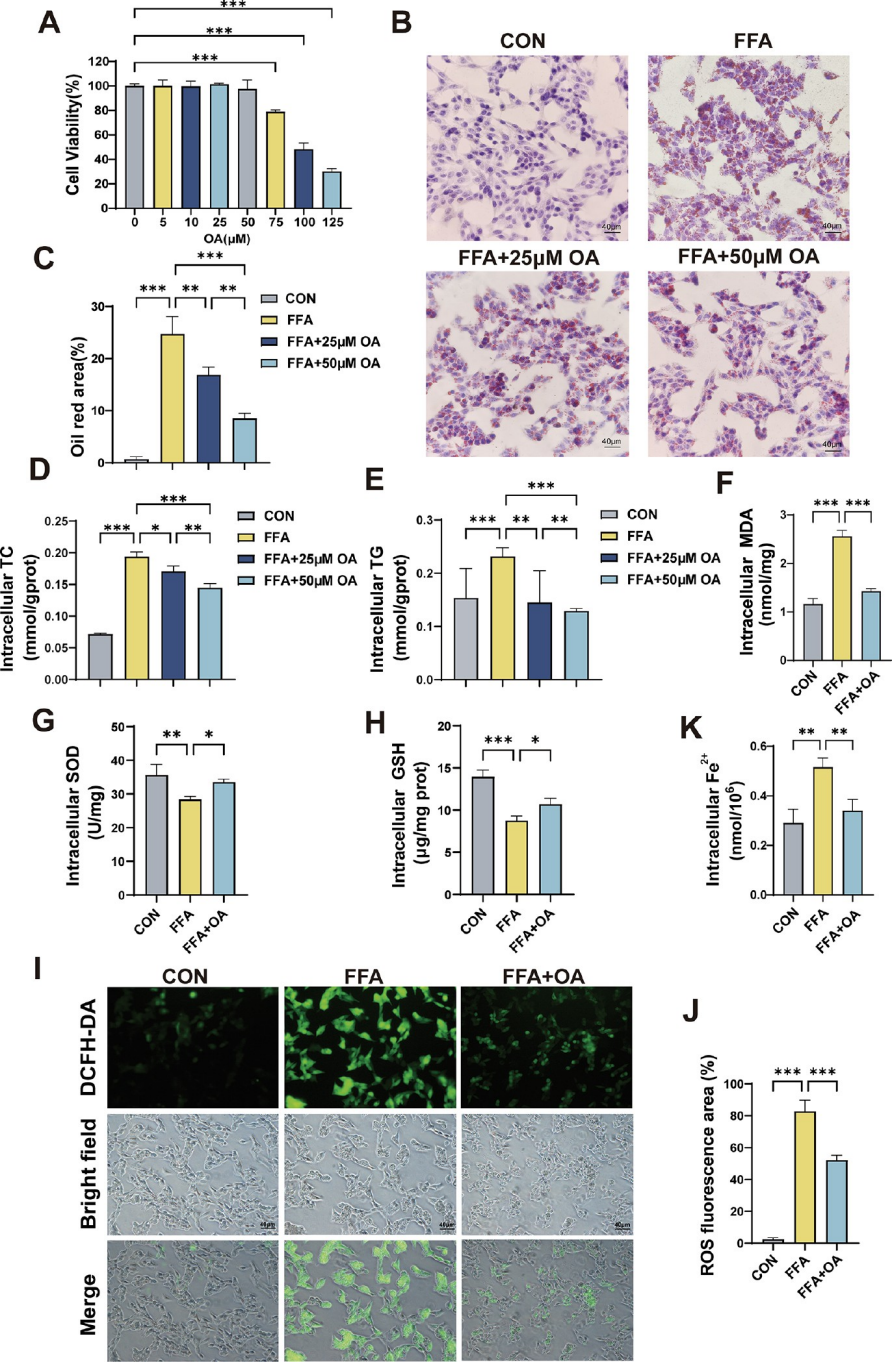
Oleanolic acid modulates intracellular lipid accumulation and oxidative stress. (A) CCK-8 assay detecting OA’s effect on HepG2 cell viability. (B–C) Intracellular Oil Red O staining and quantification. Scale bar, 40 μm. (D–E) Effects of FFA and different OA concentrations on intracellular TC and TG levels. (F–H) Effects of FFA and 50 μM OA on intracellular MDA, SOD, and GSH levels. (I–J) Intracellular ROS fluorescence staining and quantification. Scale bar, 40 μm. (K) Effects of FFA and 50 μM OA treatment on intracellular Fe^2+^ levels. Data are expressed as mean ± SD (*n* = 3). One-way ANOVA was used to assess differences among groups. *p < 0.05, **p < 0.01, and ***p < 0.001.

### 3.2 OA Modulates Cellular Oxidative Stress to Inhibit Ferroptosis

Given the close association between MASLD development and oxidative stress, we next investigated whether OA affects cellular oxidative stress levels. Our findings revealed decreased MDA levels and increased SOD and GSH levels in OA-treated cells (Figure 1F–H). As ROS serves as a reliable indicator of oxidative stress, we confirmed reduced cellular ROS levels following OA treatment via ROS fluorescence staining (Figure 1I–J). These findings confirm OA’s capacity to reduce oxidative stress. We next examined OA’s effect on intracellular Fe^2+^ levels following FFA solution treatment and found that OA treatment markedly decreased intracellular Fe^2+^ content (Figure 1K).

We examined several key ferroptosis markers. SQ-PCR analysis revealed (Figure 2A–B) that FFA solution treatment significantly downregulated the mRNA expression of ferroptosis-inhibitory genes, such as glutathione peroxidase 4 (*GPX4*), solute carrier family 7 member 11 (*SLC7A11*), and nuclear factor E2-related factor 2 (*Nrf2*), while increasing mRNA expression of the ferroptosis-promoting gene transferrin receptor 1 (*TFR1*). These mRNA expression levels were significantly reversed following OA treatment. Western blot analysis further validated these findings: the FFA group exhibited significantly reduced protein levels of GPX4, SLC7A11, and Nrf2, while TFR1 protein levels were elevated; OA treatment similarly reversed these proteins expression changes (Figure 2C–D). These consistent gene and protein expression results strongly confirm that OA treatment effectively alleviates FFA solution-induced cellular oxidative stress and suppresses ferroptosis by regulating the Nrf2/GSH/GPX4 antioxidant system and inhibiting the pro-iron uptake factor TFR1.

**Figure 2:**
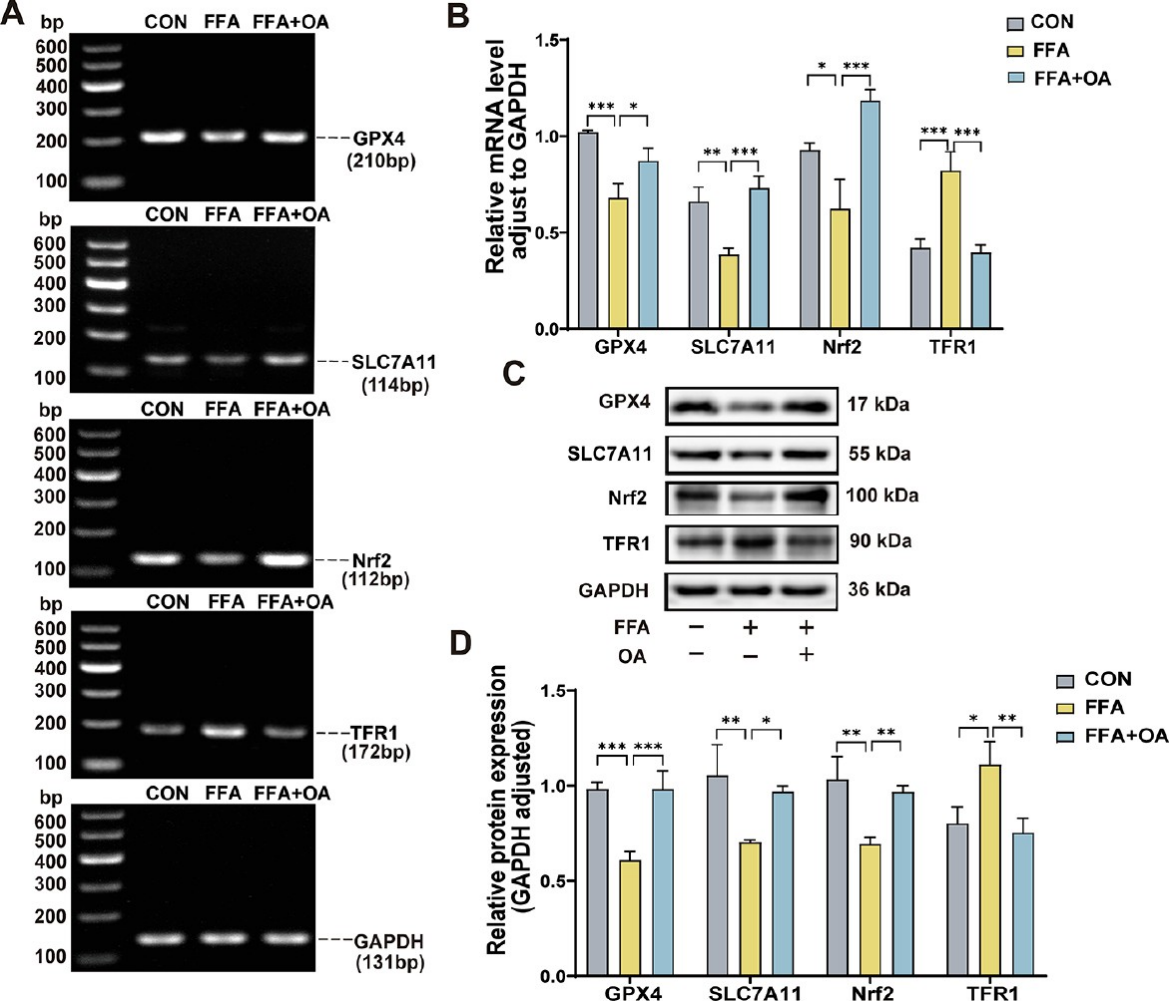
Oleanolic acid inhibits ferroptosis marker genes. (A–B) Intracellular mRNA levels of *GPX4, SLC7A11, Nrf2*, and *TFR1*. (C–D) Intracellular protein expression levels of GPX4, SLC7A11, Nrf2, and TFR1. Data are expressed as mean ± SD (*n* = 3). One-way ANOVA was used to assess differences among groups. *p < 0.05, **p < 0.01, and ***p < 0.001.

### 3.3 OA Modulates Lipid Deposition in Mice

We established a mouse model of MASLD and treated it with oral administration of oleanolic acid and the positive control drug fenofibrate, following the specific experimental protocol shown in Figure 3A. Macroscopic observations revealed that compared with the CON group, mice fed an HFD developed significant obesity; while the HFD+OA-L and HFD+OA-H groups exhibited reduced body weight relative to the HFD group, with the HFD+OA-H treatment demonstrating a more notable reduction. The livers of CON mice appeared dark red, those of HFD mice were pale yellow, whereas the liver colors of OA-treated and fenofibrate-treated mice resembled those of the CON group (Figure 3B). The mice fed a HFD showed significantly increased body weight, liver weight, and liver-to-body weight ratio. Treatment with two concentrations of OA and fenofibrate reversed these changes (Figure 3C–E). HE staining of liver sections revealed that the HFD induced abundant lipid vacuoles in the liver. Oleanolic acid treatment reduced lipid vacuoles in a dose-dependent fashion, with comparable effects to the fenofibrate. Oil Red O staining showed marked red lipid droplets in HFD mouse livers, which was significantly attenuated in all treatment arms (Figure 3F). Furthermore, HFD mice exhibited markedly higher serum and hepatic TG and TC levels, which were reduced by treatment with different doses of OA and fenofibrate (Figure 3G–J). Furthermore, the HFD group showed increased serum AST, ALT, and LDL-C levels, accompanied by reduced HDL-C. These alterations were reversed by OA and fenofibrate treatment (Figure 3K–N). These findings indicate that OA alleviates HFD-induced lipid deposition and hepatic injury.

**Figure 3:**
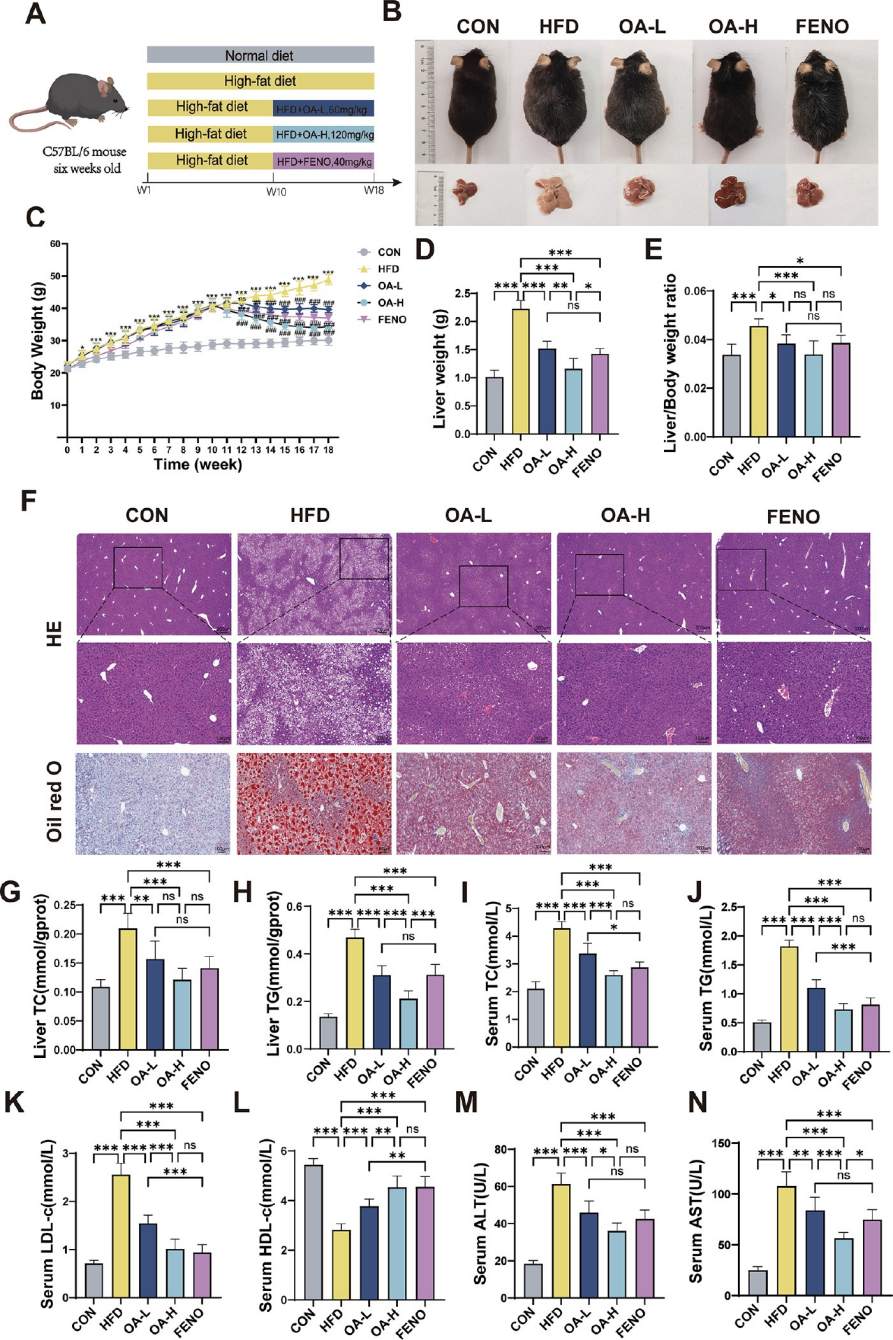
Oleanolic acid attenuates lipid accumulation in mice. (A) Schematic of the mouse experimental procedure. (B) Appearance of mice and their livers. (C–E) Changes in mouse body weight (*p < 0.05, **p < 0.01, and ***p < 0.001 vs CON group; [ns] *p* > 0.05, #*p* < 0.05, ##*p* < 0.01 and ###*p* < 0.001 vs HFD group), liver weight, and liver-to-body weight ratio. (F) Mouse liver HE staining (original scale bar: 200 μm, enlarged scale bar: 100 μm) and Oil Red O staining (scale bar: 100 μm). (G–N) Liver TC and TG content, serum TC, TG, LDL-C, HDL-C, ALT, and AST levels in mice. Data are expressed as mean ± SD (*n* = 6). One-way ANOVA was used to assess differences among groups. *p < 0.05, **p < 0.01, and ***p < 0.001.

### 3.4 OA Modulates Inflammation and Oxidative Stress in Mice and Improves Hepatic Ferroptosis

As part of our study, we investigated changes in inflammatory markers in animal serum and found that serum concentrations of IL-6 and TNF-α were significantly elevated in the HFD group, whereas these levels were reduced in the treatment arms (Figure 4A–B). We also validated changes in oxidative stress markers in the animals. The HFD group showed elevated levels of MDA and ROS in the liver, concomitantly with decreased levels of SOD and GSH (Figure 4C–F). These changes were reversed following treatment with OA and fenofibrate. Furthermore, HFD-induced accumulation of hepatic Fe^2+^ was observed, which decreased following OA and fenofibrate treatment (Figure 4G). Notably, high-dose OA demonstrated superior therapeutic effects compared to low-dose OA.

**Figure 4:**
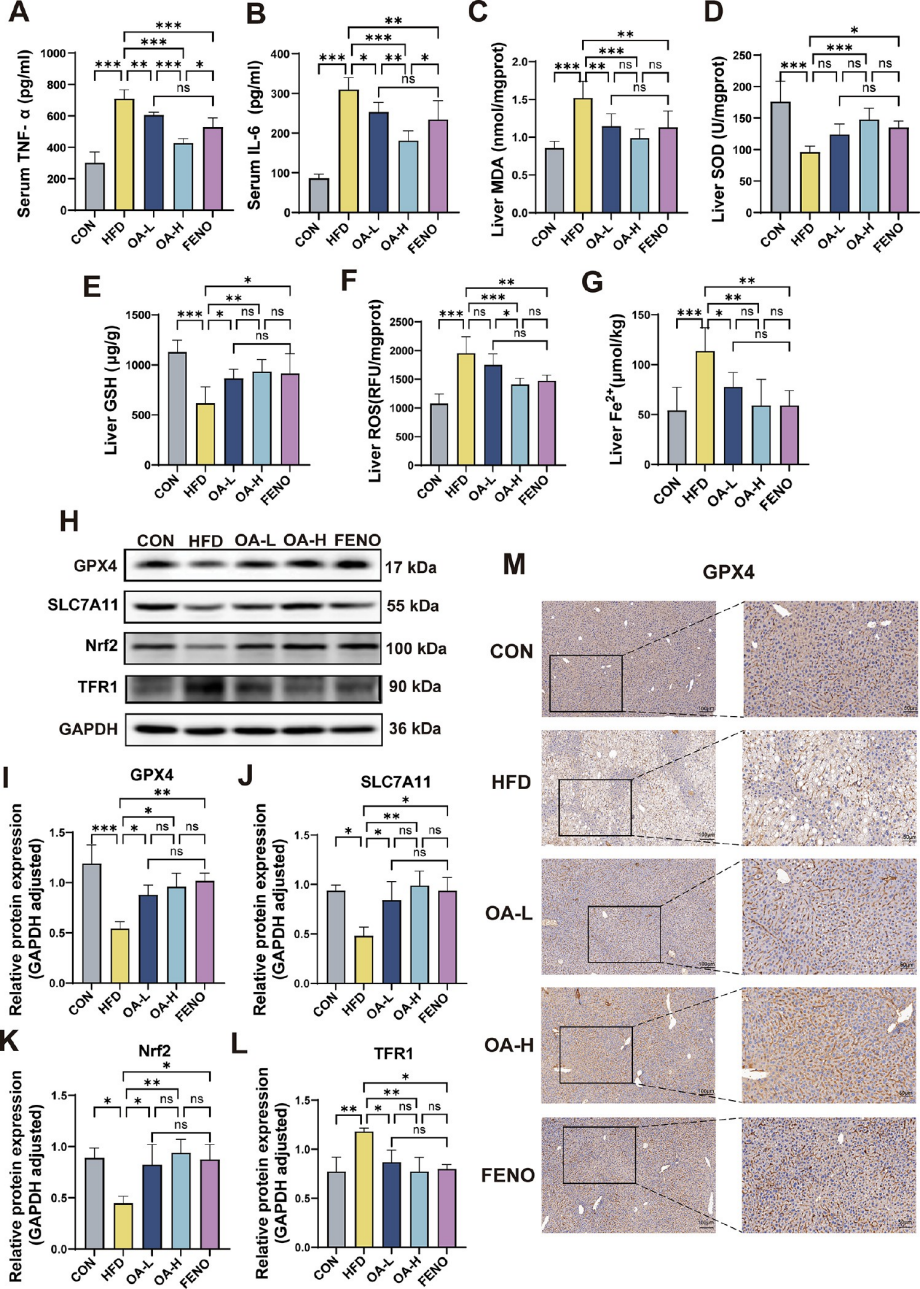
Oleanolic acid modulates inflammation and oxidative stress in mice and inhibits hepatic iron-related death. (A–B) Serum TNF-α and IL-6 levels in mice (*n* = 6). (C–G) Liver MDA, SOD, GSH, ROS, and Fe^2+^ levels in mice (*n* = 6). (H–L) Hepatic protein expression levels of GPX4, SLC7A11, Nrf2, and TFR1 (*n* = 3). (M) Hepatic IHC staining for GPX4 in mice (original scale bar: 100 μm, enlarged scale bar: 50 μm). Data are expressed as mean ± SD. One-way ANOVA was used to assess differences among groups. [ns] *p* > 0.05, *p < 0.05, **p < 0.01, and ***p < 0.001.

We further validated changes in ferroptosis markers in mice. The protein expressions of GPX4, SLC7A11, and Nrf2 were downregulated, while TFR1 was upregulated in the HFD group. These alterations were also reversed by OA and fenofibrate treatment (Figure 4H–L). Similarly, immunohistochemical results revealed significantly reduced expression of the ferroptosis marker gene GPX4 in the HFD group, which increased following OA and fenofibrate treatment (Figure 4M). These findings indicate that OA can improve inflammatory responses and oxidative stress in mice, while also mitigating ferroptosis to some extent, thereby achieving therapeutic effects against MASLD in mice.

### 3.5 Further Validation of OA-Mediated Ferroptosis Inhibition in Cells

To further investigate whether OA possesses the potential to inhibit ferroptosis, we employed Erastin—a specific activator of ferroptosis—to examine OA’s ability to suppress this process. CCK-8 assays revealed significantly reduced cell viability in cells treated with 10 μM Erastin for 24 h, whereas OA treatment mitigated Erastin-induced cell death (Figure 5A). Erastin is a classic ferroptosis inducer that impairs XCT (SLC7A11) function and GSH production. We therefore assessed changes in GSH and Fe^2+^ levels, the mRNA expression of *SLC7A11* and the ferroptosis core regulator *GPX4*, as well as their corresponding protein levels. Erastin treatment led to a notable decrease in GSH levels and a marked accumulation of intracellular Fe^2+^ (Figure 5B–C), while reducing the mRNA expression of *SLC7A11* and *GPX4* and their corresponding protein levels (Figure 5D–G). OA treatment reversed these alterations. Additionally, compared to controls, ROS-associated accumulation in Erastin-treated HepG2 cells exhibited stronger fluorescence intensity, and the ROS fluorescence signal decreased in the group treated with OA (Figure 5H–I). These findings suggest that OA may possess the potential to inhibit ferroptosis.

**Figure 5:**
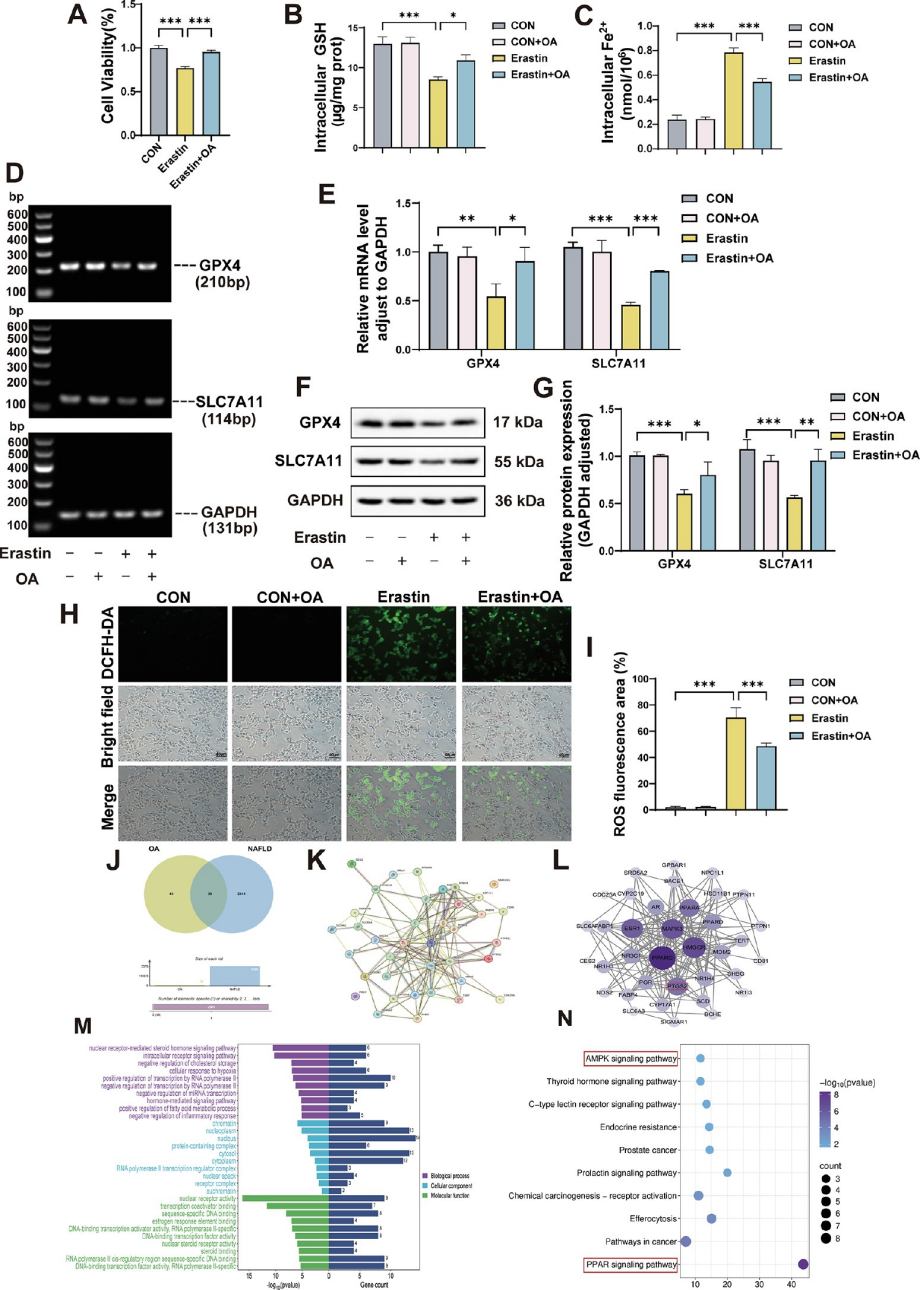
Oleanolic acid alleviates ferroptosis induced by ferroptosis activators and inhibits ferroptosis mechanisms via network pharmacology analysis. (A) Viability assay of HepG2 cells treated with Erastin and OA. (B–C) Intracellular GSH and Fe^2+^ levels in Erastin- and oleanolic acid-treated HepG2 cells. (D–E) Intracellular mRNA levels of *GPX4* and *SLC7A11*. (F–G) Intracellular protein expression levels of GPX4 and SLC7A11. (H–I) ROS fluorescence staining and quantitative analysis in HepG2 cells. Scale bar, 40 μm. (J) Venn diagram screening for shared targets between NAFLD and OA. (K) PPI network of OA’s anti-NAFLD targets. (L) Cytoscape visualization of gene interaction network; node size and color darkness reflect the degree of interaction strength. (M) Gene Ontology (GO) analysis of oleanolic acid-treated NAFLD targets. (N) Kyoto Encyclopedia of Genes and Genomes (KEGG) pathway enrichment for oleanolic acid targets in NAFLD treatment. Data are expressed as mean ± SD (*n* = 3). One-way ANOVA was used to assess differences among groups. *p < 0.05, **p < 0.01, and ***p < 0.001.

### 3.6 Targets or Pathways of OA Action Identified through Network Pharmacology Analysis

To identify OA’s potential targets for MASLD treatment, we performed a network pharmacology study. First, we identified 78 potential targets of OA via the SwissTargetPrediction database. Subsequently, 2379 MASLD-associated genes were obtained from the GeneCards database. We identified 38 overlapping genes between drug targets and disease-associated genes (Figure 5J) and visualized their interactions using a PPI network diagram (Figure 5K). Importing these genes into Cytoscape for visualization (Figure 5L), we analyzed their degree values and selected genes with degrees > median as core genes. These core genes were then subjected to GO and KEGG enrichment analysis. GO enrichment analysis revealed that target genes enriched in biological processes beneficial for treating nonalcoholic fatty liver disease primarily included negative regulation of cholesterol storage, positive regulation of fatty acid metabolism, and negative regulation of inflammatory response. These genes were located in cellular components such as chromatin, nucleoplasm, nucleus, and cytoplasm, and exhibited molecular functions including nuclear receptor activity and DNA-binding transcription factor activity (Figure 5M). KEGG results suggest that the AMPK signaling pathway and PPAR signaling pathway may be key pathways for OA treatment (Figure 5N).

### 3.7 PTGS2 Is a Key Molecular Target of OA in Inhibiting Ferroptosis

Next, we delve into the specific mechanism by which OA inhibits ferroptosis. PTGS2, identified through network pharmacology analysis as a core gene for OA treatment in MASLD, also serves as a key biomarker and effector molecule of ferroptosis. We performed semiquantitative PCR and Western blot analyses of its expression levels. In vitro experiments revealed that FFA significantly elevated intracellular PTGS2 levels, which decreased after OA administration. This phenomenon was similarly validated in vivo (Figure 6A–D): high-fat diet-induced PTGS2 levels were significantly elevated in mouse livers, and OA treatment reversed this increase (Figure 6E–F). We reasonably suspect PTGS2 as a key molecular target of OA in regulating ferroptosis. Generally, a molecular docking energy below −5 kcal/mol indicates strong binding affinity. Results showed the minimum docking energy between PTGS2 and OA was −9.4 kcal/mol, with hydrogen and hydrophobic bonds forming between them (Figure 6G–H), suggesting robust direct interaction. Subsequently, we performed molecular dynamics simulations of the OA-PTGS2 complex. Molecular dynamics simulation results show that the protein–ligand complex reached a stable equilibrium state after 40 ns of simulation, with the RMSD remaining stable at 1.7 Å. This conformational stability confirms that the small molecule exhibits good binding stability with the target protein (Figure 6I). During the simulation, the Rg value of the complex fluctuated smoothly, and no significant structural expansion or contraction was observed in the complex as a whole (Figure 6J). Analysis of the SASA indicated that ligand binding had virtually no effect on the target protein’s surface contact area, suggesting that this binding interaction had a minimal impact on the protein’s overall structural conformation (Figure 6K). Hydrogen bond statistics revealed that approximately two hydrogen bonds were consistently maintained between the ligand and the protein, ensuring stable and close intermolecular interactions (Figure 6L). The RMSF values of the vast majority of residues in the complex were below 3 Å, indicating restricted residue motion and a significant increase in overall structural rigidity (Figure 6M). The free energy landscape (FEL) plot, generated based on RMSD and Rg data, visually illustrates the energy distribution characteristics of the complex, with red representing high-energy states and blue representing low-energy, stable conformations (Figure 6N). In summary, this small molecule can stably bind to the target protein through persistent hydrogen bonding, demonstrating excellent binding affinity and structural stability. We further conducted cellular thermotranslocation assays, revealing that OA treatment significantly enhanced PTGS2 thermal stability at 60 and 65 °C (Figure 6O). These results robustly confirm the strong interaction between OA and PTGS2, demonstrating that OA inhibits ferroptosis by binding to PTGS2. Treatment with the PTGS2 activator 6-OHDA reversed the OA-induced changes: namely, the reduction in MDA levels, the increases in SOD and GSH levels, and the decrease in intracellular Fe^2+^ levels (Figure 7A–D). Meanwhile, 6-OHDA abrogated the ameliorative effect of OA on ROS accumulation (Figure 7E–F). Furthermore, 6-OHDA treatment also reversed the OA-mediated alterations in the expression of several core genes involved in ferroptosis (Figure 7G–L).

**Figure 6:**
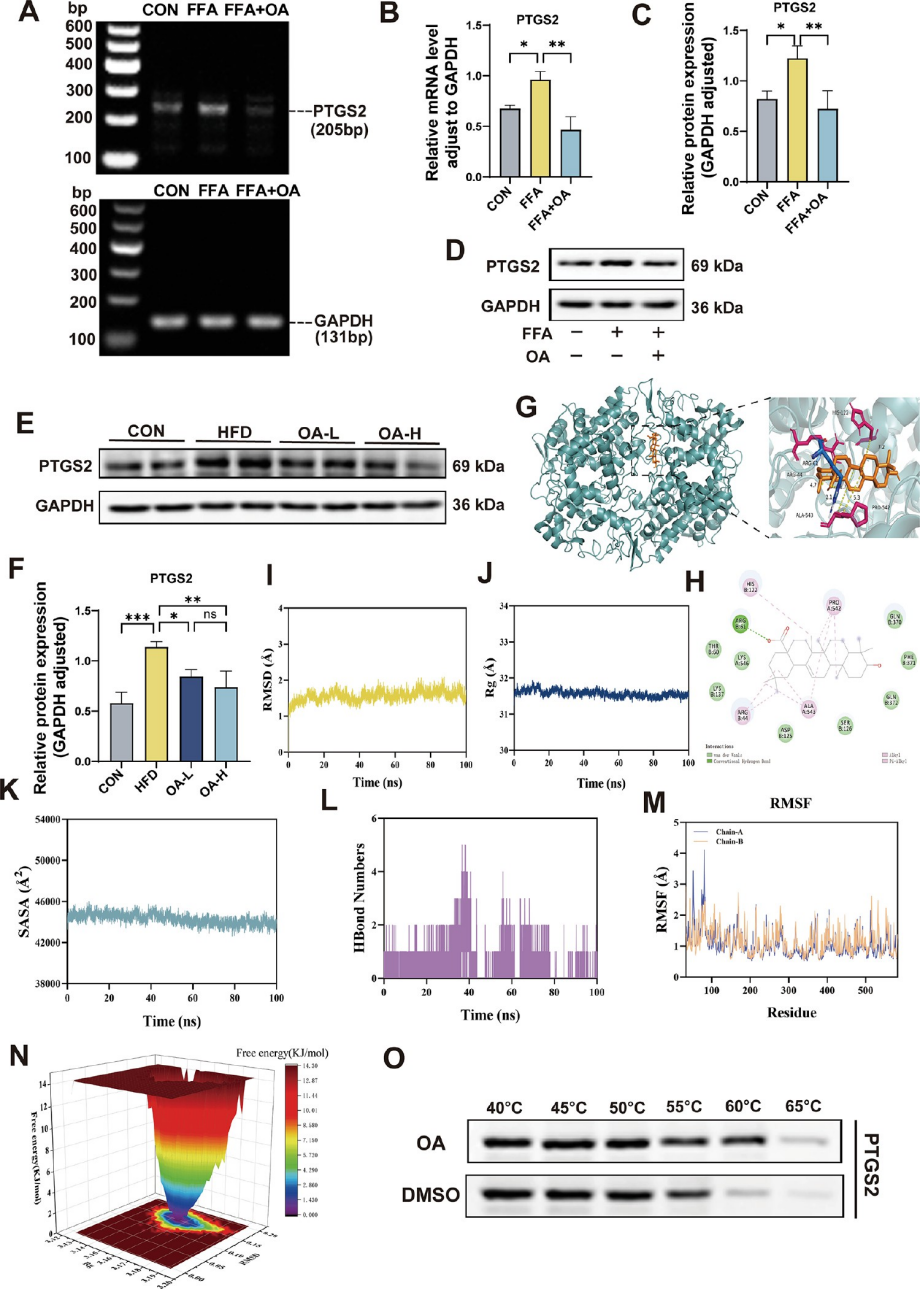
Direct binding between oleanolic acid and *PTGS2*. (A–B) Intracellular mRNA levels of *PTGS2*. (C–D) Intracellular protein expression levels of PTGS2. (E–F) PTGS2 protein expression levels in mouse livers from each group. (G–H) Molecular docking of oleanolic acid with PTGS2, 3D analysis diagram, and 2D analysis diagram. (I–M) RMSD, Rg, SASA, hydrogen bond, and RMSF trajectories of the complex over time. (N) Free energy landscape plot. (O) CETSA evaluating the effect of OA on the thermal stability of PTGS2. Data are expressed as mean ± SD (*n* = 3). One-way ANOVA was used to assess differences among groups. *p < 0.05, **p < 0.01, and ***p < 0.001.

**Figure 7:**
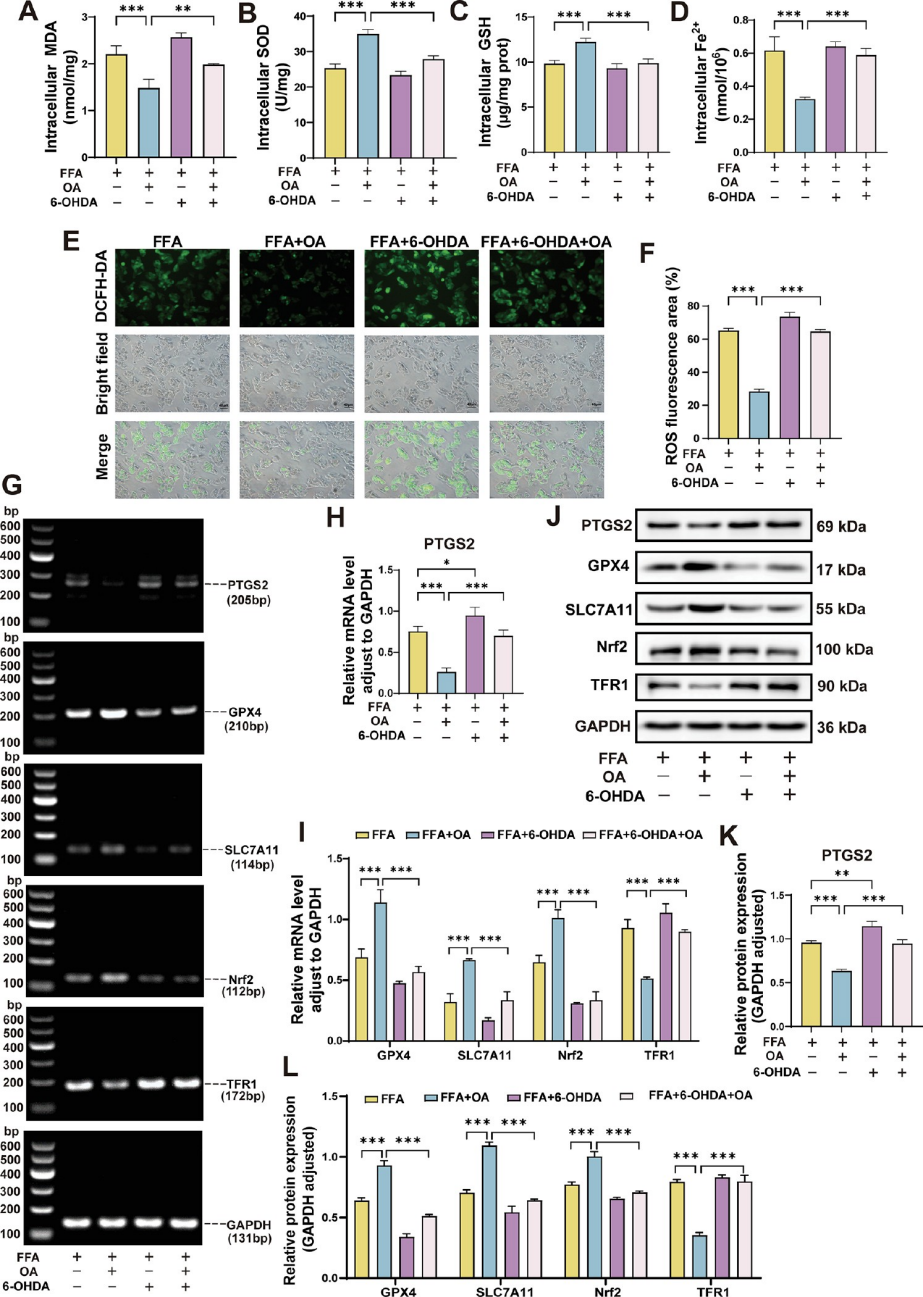
6-OHDA-mediated activation of PTGS2 negates the antiferroptotic effect of OA. (A–D) Effects of FFA, OA, and the PTGS2 activator 6-OHDA on intracellular levels of MDA, SOD, GSH, and Fe^2+^. (E–F) ROS fluorescence staining and quantitative analysis of HepG2 cells. Scale bar, 40 μm. (G–I) Semiquantitative PCR analysis and corresponding quantification of mRNA levels for *PTGS2, GPX4*, *SLC7A11, Nrf2*, and *TFR1*. (J–L) Protein expression levels of PTGS2, GPX4, SLC7A11, Nrf2, and TFR1. Data are expressed as mean ± SD (*n* = 3). One-way ANOVA was used to assess differences among groups. *p < 0.05, **p < 0.01, and ***p < 0.001.

### 3.8 OA Regulates Lipid Metabolism via the AMPK/ACC Signaling Pathway to Inhibit Ferroptosis

KEGG enrichment analysis revealed that core genes related to OA treatment were enriched in the AMPK signaling pathway. As documented in many studies, the AMPK signaling pathway exerts an inhibitory effect on ferroptosis, and the AMPK/ACC signaling pathway can regulate lipid metabolism and suppress ferroptosis.^10,34^ We validated changes in AMPK/ACC expression in mouse liver and found that OA treatment activated AMPK by phosphorylating the AMPKα subunit at site 172, which further phosphorylated ACC1 (Figure 8A–C). To further validate whether OA alters the AMPK/ACC signaling pathway, we induced HepG2 cells with the AMPK inhibitor Compound C. Similar to in vivo results, OA treatment reversed the FFA-induced decrease in AMPK and ACC phosphorylation. However, the activating effect of OA on their phosphorylation was abolished upon Compound C treatment (Figure 8G–I). Semiquantitative PCR results also showed corresponding changes (Figure 8D–F). Notably, these core ferroptosis genes also reversed the OA-induced changes under Compound C treatment (Figure 9A–D). Oil red staining confirmed that Compound C blocked OA’s lipid droplet-reducing effect (Figure 9E–F). Additionally, TC and TG measurements indicated Compound C impaired OA’s suppression of lipid accumulation (Figure 9G–H). Consistently, Compound C treatment also blocked OA’s improvement of oxidative stress. The FFA+OA group displayed lower MDA and increased SOD and GSH levels, compared to the FFA group. These beneficial effects of OA were abolished in the FFA+OA+Compound C group (Figure 9I–K). Moreover, Compound C treatment reversed OA’s improvement in ROS accumulation (Figure 9L–M) and blocked OA’s therapeutic effects in reducing Fe^2+^ levels and improving iron deposition (Figure 9N). Collectively, these results indicate that OA inhibits ferroptosis by regulating lipid metabolism through the AMPK/ACC signaling pathway.

**Figure 8:**
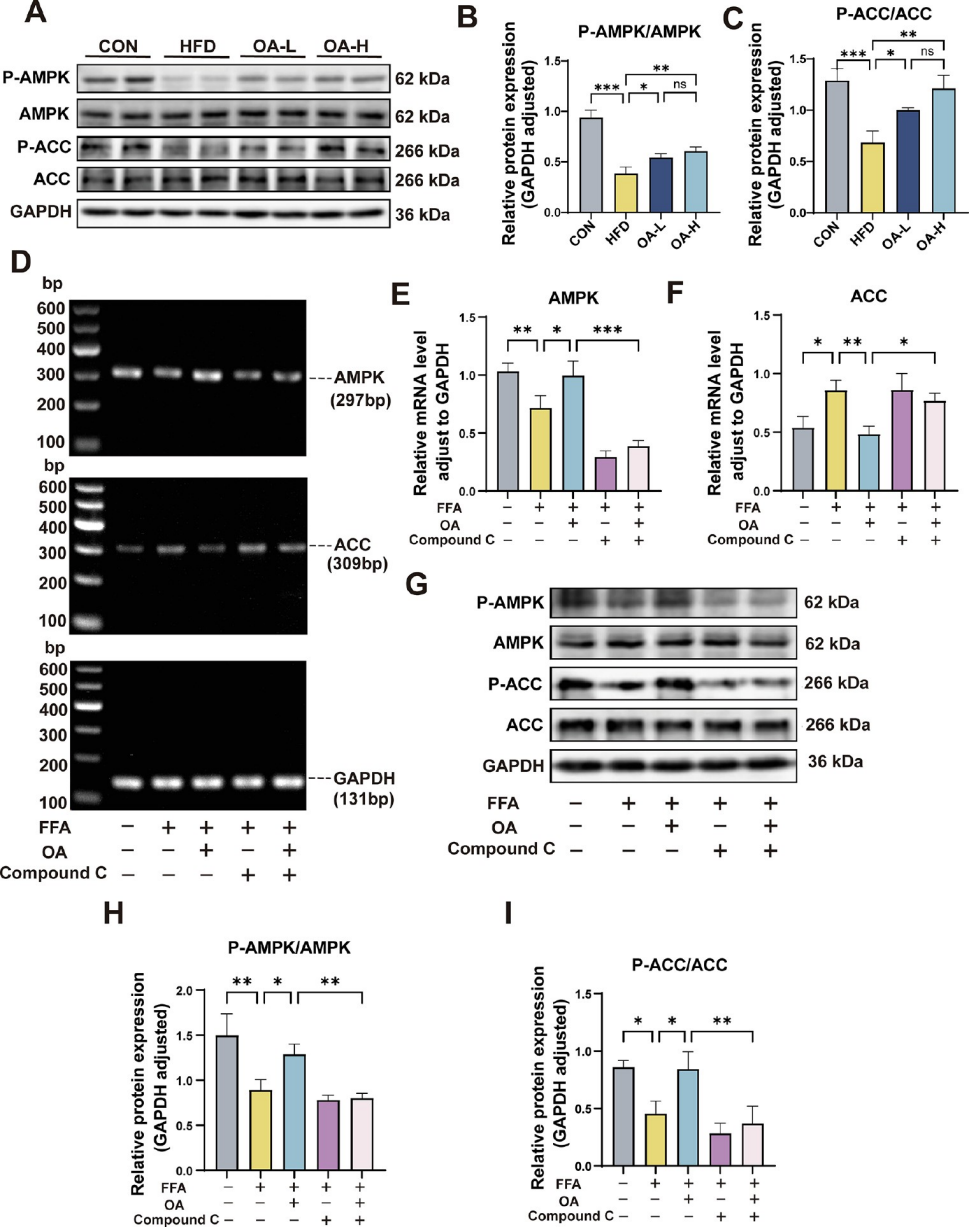
Oleanolic acid activates the AMPK/ACC signaling pathway. (A–C) AMPK-α, phospho-AMPK-α (Thr172), ACC1, and phospho-ACC1 (S79) protein expression levels in the livers of mice. (D–F) Intracellular mRNA levels of *AMPK* and *ACC* in CON, FFA, FFA+OA, FFA+Compound C, and FFA+Compound C+OA groups. (G–I) Western blot analysis of AMPK-α, phospho-AMPK-α (Thr172), ACC1, and phospho-ACC1 (S79) protein expression levels in HepG2 cells from different groups. Data are expressed as mean ± SD (*n* = 3). One-way ANOVA was used to assess differences among groups. *p < 0.05, **p < 0.01, and ***p < 0.001.

**Figure 9:**
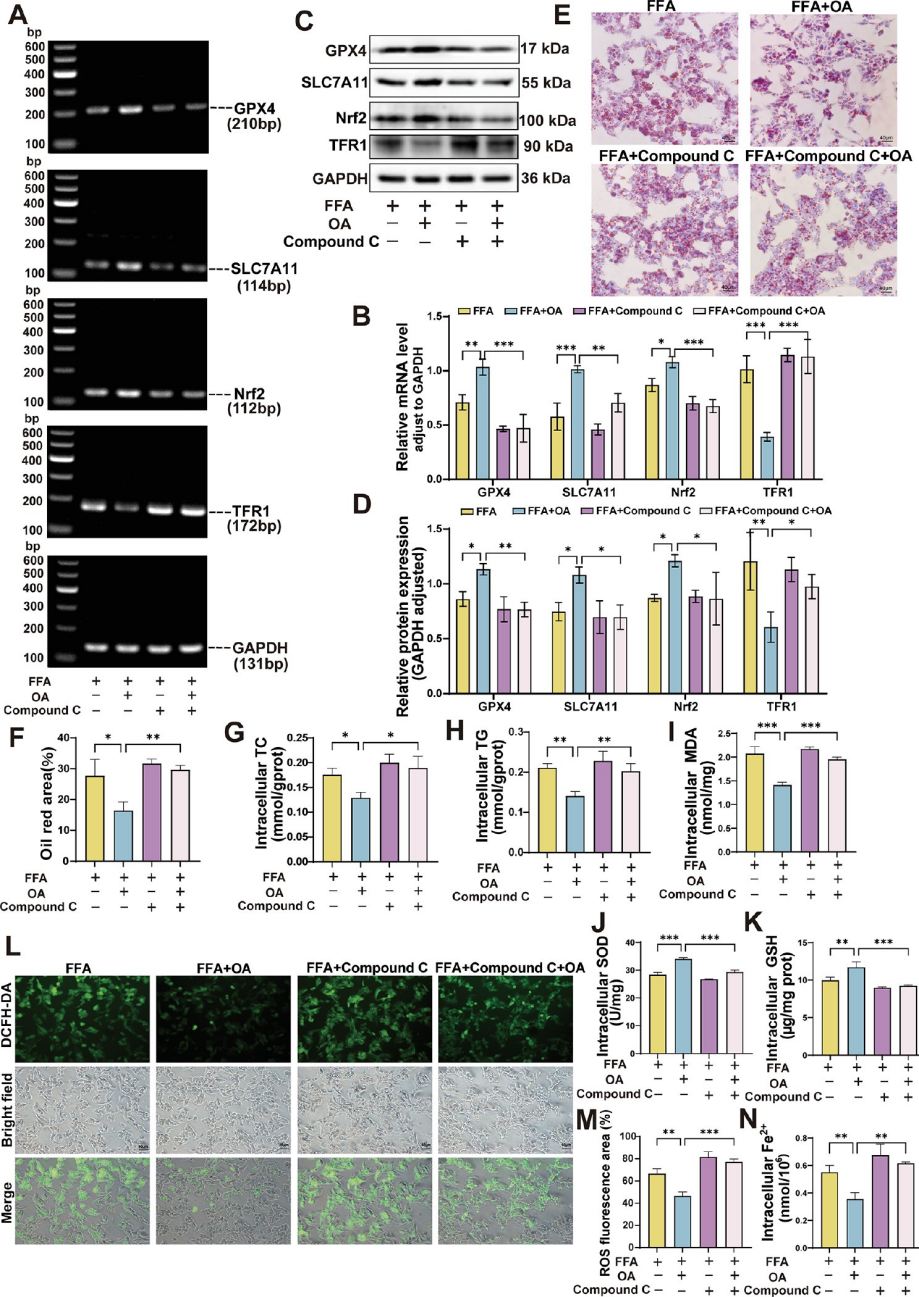
AMPK inhibitor-mediated validation of AMPK/ACC axis antilipotoxicity and ferroptosis inhibition. (A–B) Intracellular mRNA levels of *GPX4, SLC7A11, Nrf2*, and *TFR1* in FFA, FFA+OA, FFA+Compound C, and FFA+Compound C+OA groups. (C–D) Intracellular protein expression levels of GPX4, SLC7A11, Nrf2, and TFR1. (E–F) Intracellular Oil Red O staining and quantification. (G–K) Intracellular levels of TC, TG, MDA, SOD, and GSH. (L–M) Intracellular ROS fluorescence staining and quantitative analysis. Scale bar, 40 μm. (N) Intracellular Fe^2+^ levels. Data are expressed as mean ± SD (*n* = 3). One-way ANOVA was used to assess differences among groups. *p < 0.05, **p < 0.01, and ***p < 0.001.

## 4 Discussion

MASLD poses a major global public health challenge, with its rising incidence closely associated with metabolic syndromes including obesity, insulin resistance, and dyslipidemia.^35–37^ Its pathophysiological mechanisms have been extensively studied, encompassing lipid metabolism disorders, oxidative stress, inflammatory responses, and cell death.^38^ However, to date, there remains a lack of safe and approved effective drug interventions in clinical practice, highlighting the urgency of seeking novel therapeutic strategies. Natural products have attracted considerable interest in MASLD treatment research due to their multitargeted and low-toxicity advantages. Based on the FDA-recommended method for converting body surface area,^39^ the oral doses of oleanolic acid (OA) administered to C57BL/6 mice in this study (60 mg/kg and 120 mg/kg) translate to human equivalent doses (HED) of approximately 291.6 mg per day and 583.8 mg per day, respectively. Existing literature^40^ clearly indicates that the recommended dosage of oleanolic acid in dietary supplements is typically 100–500 mg/day, with an estimated safety margin of 7.5–47.8 times the recommended dose. The human equivalent doses calculated in this study (291.6–583.8 mg/day) fall within this recommended safety margin. Furthermore, no significant toxicological responses were observed in animals administered the compound continuously for 8 weeks. Combined with oleanolic acid’s status as a naturally occurring, edible plant-derived compound, these findings suggest the doses used in this study possess favorable clinical translation feasibility and safety. We evaluated the therapeutic efficacy of the natural compound OA and compared it with the clinically established positive control drug fenofibrate. Fenofibrate, a commonly used PPAR-α agonist, effectively regulates lipid catabolism and removes abnormal blood lipids, serving as a classic drug targeting the core metabolic disorder (dyslipidemia) in NAFLD.^41^ Furthermore, existing research supports fenofibrate’s ability to modulate ferroptosis.^42–44^ This study confirms that the natural triterpenoid compound OA significantly reduces lipid accumulation and damage in both HFD-induced MASLD mice and hepatocytes. More importantly, we first demonstrate that OA’s hepatoprotective effects are closely linked to its inhibition of ferroptosis—a novel iron-dependent type of cell death—and preliminarily reveal its molecular mechanism involving regulation of the AMPK/ACC and PTGS2 signaling axes.

Ferroptosis, an iron-dependent programmed cell death triggered via lipid peroxidation, has been identified as a key mechanism driving MASLD/MASH progression.^45^ Our findings further confirm the close association between ferroptosis and MASLD, revealing that oleanolic acid (OA) exerts therapeutic effects by inhibiting ferroptosis. In liver tissues of MASLD model mice and in vitro cellular models, we observed pronounced ferroptosis hallmarks:^46,47^ The lipid metabolism imbalance in MASLD serves as a core driver. It induces endoplasmic reticulum stress, elevates IL-6 and TNF-α levels in mice, and triggers persistent inflammation. This excessive inflammatory response further leads to excessive accumulation of ROS, thereby inducing severe oxidative stress—the core pathological basis for hepatocyte injury and ferroptosis initiation. Both in vivo and cellular assays demonstrated significantly elevated levels of MDA and ROS, reflecting the extent of excessive lipid oxidation. Concurrently, key components of the antioxidant defense system protecting cellular structure and function, such as GSH and SOD activity, showed marked depletion, indirectly indicating the exhaustion of hepatocytes’ capacity to counteract oxidative damage. Moreover, abnormal accumulation of ferrous ions (Fe^2+^) provided catalytic support for iron-dependent lipid peroxidation. These findings collectively confirm ferroptosis occurrence in both the HFD-fed in vivo model and the FFA-induced in vitro model. Following oleanolic acid administration, all aforementioned biochemical indicators were significantly improved, indicating effective suppression of lipid peroxidation and iron accumulation. A hallmark of ferroptosis is intracellular iron accumulation accompanied by uncontrolled lipid peroxidation. The experiments confirmed impaired function of the light chain subunit SLC7A11 of the cystine/glutamate antiporter System Xc^–^ in the model group, leading to restricted synthesis and depletion of the intracellular antioxidant GSH. GSH depletion reduces or inactivates the crucial antioxidant enzyme GPX4, preventing clearance of toxic lipid peroxides. This triggers catastrophic excessive buildup of ROS and generation of lipid radicals.^48–50^ Concurrently, increased iron uptake mediated by transferrin receptor 1 (TFR1) in mouse liver and HepG2 cells exacerbates intracellular accumulation of free iron (Fe^2+^).^51^ This provides a catalyst for lipid peroxidation. Downregulation of Nrf2 in the modeling group indicates impaired antioxidant function, as Nrf2 is a key transcription factor regulating antioxidant systems including the Nrf2/GSH/GPX4 axis^52^ by upregulating antioxidant gene expression, playing a crucial protective role in resisting hepatic ferroptosis. Western blot and PCR analyses revealed that OA significantly increased GPX4, SLC7A11, and Nrf2 expression while downregulating TFR1, thereby restoring cellular antioxidant capacity and iron homeostasis. To further emphasize OA’s direct inhibitory effect on ferroptosis, we introduced the classic ferroptosis activator Erastin. Erastin treatment significantly induced ferroptosis in vitro, manifested by abnormal accumulation of ROS and Fe^2+^, depletion of GSH, and dramatic downregulation of key inhibitory molecules such as GPX4 and SLC7A11. However, this damage was completely reversed by OA pretreatment. These findings collectively indicate that inhibiting ferroptosis in hepatocytes is a key pathway by which OA alleviates MASLD.

To elucidate the mechanism by which oleanolic acid inhibits ferroptosis, we combined network pharmacology with experimental validation, focusing on two signaling axes: PTGS2 and AMPK/ACC. Through network pharmacology analysis, we identified PTGS2 as one of the core targets regulated by oleanolic acid in modulating ferroptosis and MASLD. PTGS2 (Cyclooxygenase-2), also known as COX-2, serves not only as a key rate-limiting enzyme in inflammatory responses but also as a crucial executor of lipid peroxidation within the ferroptosis pathway. The essence of ferroptosis lies in the uncontrolled oxidation of PUFAs on membrane phospholipids. PTGS2 directly catalyzes the conversion of PUFAs like arachidonic acid (AA) into highly pro-inflammatory and pro-oxidative lipid products (e.g., precursors of prostaglandin PGE_2_), thereby accelerating membrane lipid peroxidation and directly driving ferroptosis.^53^ Abnormal PTGS2 upregulation in MASLD not only signifies severe inflammatory injury but also directly links inflammatory signaling to the pathological process of cell death.^9^ Our research demonstrates through molecular docking, MD simulations, and CETSA that oleanolic acid can directly bind to the PTGS2 protein, potentially representing a key mechanism for its pharmacological effects. In vivo studies confirm significantly elevated PTGS2 expression in the livers of MASLD mice, accompanied by increased levels of inflammatory mediators including IL-6 and TNF-α. Oleanolic acid treatment markedly suppressed both PTGS2 expression and activity. This suggests that oleanolic acid (OA) may exert its core anti-inflammatory and lipid peroxidation chain reaction-blocking (“execution-blocking”) effects by targeting PTGS2 as a key molecular target, thereby effectively mitigating hepatic oxidative damage and inflammatory burden—representing a precise strike against the ferroptosis execution pathway.

On the other hand, the AMPK/ACC pathway serves as a central regulator of cellular lipid metabolism. This study demonstrates that OA significantly activates AMPK, promoting the phosphorylation and inactivation of its downstream target ACC. ACC inactivation simultaneously suppresses de novo fatty acid synthesis, reducing the supply of PUFAs available for lipid peroxidation at the source. Concurrently, it alleviates inhibition of fatty acid β-oxidation by lowering malonyl-CoA levels, accelerating lipid clearance.^54,55^ Through this dual mechanism of “suppression and promotion,” OA significantly reduces lipid accumulation and toxicity within hepatocytes. We define this strategy of reducing oxidizable substrate supply by regulating lipid synthesis and degradation as “source restriction” against ferroptosis. This “source restriction” strategy provides a metabolic basis for resisting ferroptosis. Experiments using the AMPK inhibitor Compound C confirmed that blocking this pathway significantly weakened OA’s protective effects, emphasizing the necessity of the AMPK/ACC axis in OA’s efficacy.

Based on these findings, we propose an integrated model: OA synergistically defends hepatocytes against ferroptosis through dual mechanisms. On one hand, it activates the AMPK/ACC pathway to remodel lipid metabolic homeostasis, reducing the generation of ferroptosis-promoting lipid substrates (“source restriction”). On the other hand, it inhibits the expression of the PTGS2 molecule, blocking the peroxidation chain reaction of existing lipids and inflammatory amplification (“execution blockade”). The synergistic action of these two pathways likely constitutes OA’s potent cellular protective network in MASLD. However, it must be pointed out that the current experimental data primarily demonstrate the parallel regulatory effects of oleanolic acid (OA) on both PTGS2 and AMPK/ACC targets; this study has not directly confirmed the existence of signal crosstalk or interdependence between PTGS2 inhibition and AMPK activation. Therefore, the proposed “dual-axis synergistic mechanism” currently serves merely as a conceptual working model, representing a reasonable inference based on parallel experimental evidence rather than a conclusively demonstrated interaction. Based on existing literature, we hypothesize that potential signal crosstalk between these two pathways may occur through the following two primary modes, thereby providing a scientific theoretical basis for their synergistic effects. First, previous studies^56,57^ suggest that AMPK exerts anti-inflammatory and antiferroptotic effects by regulating downstream signaling networks, thereby suppressing the expression of PTGS2 (COX-2). For instance, recent studies have demonstrated that pharmacological activation of AMPK significantly downregulates PTGS2 expression to mitigate ferroptosis, whereas the blockade or genetic depletion of AMPK abolishes this PTGS2-inhibitory effect. Similarly, it has been confirmed^58,59^ that direct activation of AMPK by AICAR can reverse COX-2 overexpression. Thus, the OA-induced activation of AMPK may indirectly facilitate the suppression of PTGS2 at the transcriptional level. Second, the excessive accumulation of lipid peroxides and pro-inflammatory mediators driven by PTGS2 activity exacerbates cellular oxidative stress,^46,60^ and severe oxidative stress has been shown to impair the sensitivity and function of cellular energy sensors such as AMPK.^61,62^ The direct binding and inhibition of PTGS2 by OA can effectively alleviate this oxidative stress-induced pathway blockade, thereby creating a highly permissive cellular metabolic microenvironment for maintaining sustained AMPK activation. In summary, whether these two pathways act parallelly as independent targets of OA or are intrinsically coupled through the aforementioned mechanisms remains to be further validated by future experiments, such as observing the impact of OA on the AMPK pathway under specific conditions like PTGS2 deficiency.

This study reveals a novel mechanism by which oleanolic acid alleviates MASLD by inhibiting hepatic ferroptosis through dual regulation of AMPK/ACC and PTGS2 signaling. This discovery offers fresh perspectives regarding the multitarget therapeutic potential of natural compounds for metabolic liver diseases. Nevertheless, the study has several limitations: First, this study did not directly assess the enzymatic catalytic activity of PTGS2 or the generation of its downstream metabolites, thus failing to confirm whether OA directly inhibits PTGS2 enzyme function. Our findings only support that OA modulates PTGS2 expression through physical binding, thereby exerting antiferroptotic and anti-inflammatory effects. The direct binding of OA to PTGS2 and its functional consequences require further biochemical experiments (such as site-directed mutagenesis) for definitive confirmation. Second, the interaction between the AMPK/ACC and PTGS2 pathways remains unelucidated. Third, the study was primarily conducted in preclinical models, and the long-term safety of OA and its efficacy in human MASLD require further evaluation. Future work will address these aspects to advance OA as a potential therapeutic candidate for MASLD.

In summary, this study comprehensively elucidates the therapeutic mechanism of OA in MASLD. We confirmed oleanolic acid exerts protective effects through a dual mechanism: first, it targets PTGS2 as a key molecule to effectively suppress hepatic inflammation and lipid peroxidation; second, it activates the AMPK/ACC signaling pathway to mitigate lipid toxicity at the metabolic source, restore cellular antioxidant capacity and iron homeostasis, ultimately inhibiting ferroptosis and improving MASLD pathology. Such outcomes not only expand current insights into the linkage between MASLD and ferroptosis but also provide robust molecular mechanisms and preclinical evidence supporting oleanolic acid as a natural therapeutic agent for MASLD.

## Supplementary Material



## Notes

Composition of high-fat diet (Table S1), database URLs (Table S2), sequences for SQ-PCR primers (Table S3), and antibodies and reagents used for Western blot analysis (Table S4) (PDF)

## References

[1] Miao L., Targher G., Byrne C. D., Cao Y. Y., Zheng M. H. (2024). Current
status and future trends of the global burden of MASLD. Trends Endocrinol. Metab..

[2] Han S. K., Baik S. K., Kim M. Y. (2023). Non-alcoholic
fatty liver disease:
Definition and subtypes. Clin Mol. Hepatol..

[3] Byrne C. D., Targher G. (2015). NAFLD: A multisystem disease. J. Hepatol..

[4] Huang D. Q., El-Serag H. B., Loomba R. (2021). Global epidemiology
of NAFLD-related
HCC: trends, predictions, risk factors and prevention. Nat. Rev. Gastroenterol. Hepatol..

[5] Chen J., Li X., Ge C., Min J., Wang F. (2022). The multifaceted role
of ferroptosis in liver disease. Cell Death
Differ..

[6] Li S., Zhang G., Hu J., Tian Y., Fu X. (2024). Ferroptosis
at the nexus of metabolism and metabolic diseases. Theranostics.

[7] Tang D., Chen X., Kang R., Kroemer G. (2021). Ferroptosis: molecular
mechanisms and health implications. Cell Res..

[8] Zhang L., Hou N., Chen B., Kan C., Han F., Zhang J., Sun X. (2022). Post-Translational Modifications of p53 in Ferroptosis: Novel Pharmacological
Targets for Cancer Therapy. Front. Pharmacol..

[9] Jin X., Tang J., Qiu X., Nie X., Ou S., Wu G., Zhang R., Zhu J. (2024). Ferroptosis:
Emerging mechanisms,
biological function, and therapeutic potential in cancer and inflammation. Cell Death Discovery.

[10] Lee H., Zandkarimi F., Zhang Y., Meena J. K., Kim J., Zhuang L., Tyagi S., Ma L., Westbrook T. F., Steinberg G. R. (2020). Energy-stress-mediated AMPK activation inhibits
ferroptosis. Nat. Cell Biol..

[11] Liu Y., Zhan J., Wang J., Zeng X., Liu S., Huang L., Niu L., Sun C., Ding Z., Xing Y. (2025). Ferroptosis: A double-edged
sword that enhances radiation
sensitivity and facilitates radiation-induced injury in tumors. Front. Immunol..

[12] Yan H.-F., Zou T., Tuo Q.-Z., Xu S., Li H., Belaidi A. A., Lei P. (2021). Ferroptosis: Mechanisms
and links with diseases. Signal Transduction
Targeted Ther..

[13] Pollier J., Goossens A. (2012). Oleanolic acid. Phytochemistry.

[14] Verma N., Raghuvanshi D. S., Singh R. V. (2024). Recent advances in the chemistry
and biology of oleanolic acid and its derivatives. Eur. J. Med. Chem..

[15] Günther A., Bednarczyk-Cwynar B. (2025). Oleanolic Acid: A Promising Antioxidant-Sources, Mechanisms
of Action, Therapeutic Potential, and Enhancement of Bioactivity. Antioxidants.

[16] Silva F. S., Oliveira P. J., Duarte M. F. O. (2016). Ursolic,
and Betulinic Acids as Food
Supplements or Pharmaceutical Agents for Type 2 Diabetes: Promise
or Illusion?. J. Agric. Food Chem..

[17] Christodoulou A., Nikolaou P. E., Symeonidi L., Katogiannis K., Pechlivani L., Nikou T., Varela A., Chania C., Zerikiotis S., Efentakis P. (2024). Cardioprotective potential
of oleuropein, hydroxytyrosol, oleocanthal and their combination:
Unravelling complementary effects on acute myocardial infarction and
metabolic syndrome. Redox Biol..

[18] Chen C., Ai Q., Shi A., Wang N., Wang L., Wei Y. (2023). Oleanolic
acid and ursolic acid: therapeutic potential in neurodegenerative
diseases, neuropsychiatric diseases and other brain disorders. Nutr. Neurosci..

[19] Shanmugam M. K., Dai X., Kumar A. P., Tan B. K., Sethi G., Bishayee A. (2014). Oleanolic
acid and its synthetic derivatives for the prevention and therapy
of cancer: preclinical and clinical evidence. Cancer Lett..

[20] Xue C., Li Y., Lv H., Zhang L., Bi C., Dong N., Shan A., Wang J. (2021). Oleanolic Acid Targets
the Gut-Liver
Axis to Alleviate Metabolic Disorders and Hepatic Steatosis. J. Agric. Food Chem..

[21] Zhang G., Zhang H., Dong R., Zhao H., Li J., Yue W., Ma Z. (2024). Oleanolic
acid attenuates obesity through modulating
the lipid metabolism in high-fat diet-fed mice. Food Sci. Nutr.

[22] Guan Q., Wang Z., Hu K., Cao J., Dong Y., Chen Y. (2023). Melatonin Ameliorates Hepatic Ferroptosis
in NAFLD by Inhibiting
ER Stress via the MT2/cAMP/PKA/IRE1 Signaling Pathway. Int. J. Biol. Sci..

[23] Liu T., Wang J., Tong Y., Wu L., Xie Y., He P., Lin S., Hu X. (2024). Integrating
network pharmacology
and animal experimental validation to investigate the action mechanism
of oleanolic acid in obesity. J. Transl. Med..

[24] Wang X., Wang J., Ying C., Xing Y., Su X., Men K. (2024). Fenofibrate alleviates
NAFLD by enhancing the PPARα/PGC-1α
signaling pathway coupling mitochondrial function. BMC Pharmacol. Toxicol..

[25] Pan J., Yang C., Xu A., Zhang H., Fan Y., Zeng R., Chen L., Liu X., Wang Y. (2024). Salusin-α
alleviates lipid metabolism disorders via regulation of the downstream
lipogenesis genes through the LKB1/AMPK pathway. Int. J. Mol. Med..

[26] Yin X., Liu Z., Li C., Wang J. (2025). Hinokitiol ameliorates MASH in mice
by therapeutic targeting of hepatic Nrf2 and inhibiting hepatocyte
ferroptosis. Phytomedicine.

[27] Stelzer G., Rosen N., Plaschkes I., Zimmerman S., Twik M., Fishilevich S., Stein T. I., Nudel R., Lieder I., Mazor Y., Kaplan S. (2016). The GeneCards
Suite: From Gene Data Mining to Disease Genome Sequence Analyses. Curr. Protoc. Bioinf..

[28] Kim S., Chen J., Cheng T., Gindulyte A., He J., He S., Li Q., Shoemaker B. A., Thiessen P. A., Yu B. (2021). PubChem in 2021: New
data content and improved web interfaces. Nucleic
Acids Res..

[29] Gfeller D., Grosdidier A., Wirth M., Daina A., Michielin O., Zoete V. (2014). SwissTargetPrediction: A web server for target prediction of bioactive
small molecules. Nucleic Acids Res..

[30] Szklarczyk D., Nastou K., Koutrouli M., Kirsch R., Mehryary F., Hachilif R., Hu D., Peluso M. E., Huang Q., Fang T. (2025). The STRING database in 2025: Protein networks with
directionality of regulation. Nucleic Acids
Res..

[31] Ono K., Fong D., Gao C., Churas C., Pillich R., Lenkiewicz J., Pratt D., Pico A. R., Hanspers K., Xin Y. (2025). Cytoscape Web: Bringing network biology to the browser. Nucleic Acids Res..

[32] Sherman B. T., Hao M., Qiu J., Jiao X., Baseler M. W., Lane H. C., Imamichi T., Chang W. (2022). DAVID: a web server for functional
enrichment analysis and functional annotation of gene lists (2021
update). Nucleic Acids Res..

[33] Mark P., Nilsson L. (2001). Structure and Dynamics
of the TIP3P, SPC, and SPC/E
Water Models at 298 K. J. Phys. Chem. A.

[34] Li C., Dong X., Du W., Shi X., Chen K., Zhang W., Gao M. (2020). LKB1-AMPK axis negatively
regulates
ferroptosis by inhibiting fatty acid synthesis. Signal Transduction Targeted Ther..

[35] Younossi Z. M., Golabi P., Paik J. M., Henry A., Van Dongen C., Henry L. (2023). The global epidemiology of nonalcoholic
fatty liver disease (NAFLD)
and nonalcoholic steatohepatitis (NASH): a systematic review. Hepatology.

[36] Åberg F., Byrne C. D., Pirola C. J., Männistö V., Sookoian S. (2023). Alcohol consumption
and metabolic syndrome: Clinical
and epidemiological impact on liver disease. J. Hepatol..

[37] Muzurović E., Mikhailidis D. P., Mantzoros C. (2021). Non-alcoholic
fatty liver disease,
insulin resistance, metabolic syndrome and their association with
vascular risk. Metabolism.

[38] Bessone F., Razori M. V., Roma M. G. (2019). Molecular pathways
of nonalcoholic
fatty liver disease development and progression. Cell. Mol. Life Sci..

[39] Nair A. B., Jacob S. (2016). A simple practice guide
for dose conversion between animals and human. J. Basic Clin Pharm..

[40] Ray, S. D. ; Krmic, M. ; Hussain, A. ; Marvilli, C. ; Fabian, R. ; Niha, A. ; Danai, M. ; Smith, Z. ; Jalshgari, A. ; Malik, N. , Toxicity of natural products. In Encyclopedia of Toxicology (Fourth ed.); Academic Press: 2024, pp. 257–282.

[41] Qiu Y. Y., Zhang J., Zeng F. Y., Zhu Y. Z. (2023). Roles of the peroxisome
proliferator-activated receptors (PPARs) in the pathogenesis of nonalcoholic
fatty liver disease (NAFLD). Pharmacol. Res..

[42] Liu W., Zhou X., Xiao L., Huang X., Chang D., Zhong X., Zeng M., Xian Y., Zheng Y., Huang W. (2025). The gut
microbiota-mediated ferroptosis pathway: A
key mechanism of ginsenoside Rd against metabolism-associated fatty
liver disease. Chin Med..

[43] Li S., Zheng L., Zhang J., Liu X., Wu Z. (2021). Inhibition
of ferroptosis by up-regulating Nrf2 delayed the progression of diabetic
nephropathy. Free Rad. Biol. Med..

[44] Zhang G., Zhao L., Wang J., Wang K., Ji X., Hu R., Hou T., Zhang L., Li R., Sun Q. (2025). Effects
of extreme heat exposure on heatstroke and liver injury in
mice: The role of PPARα. Environ. Health
Perspect..

[45] Xu H.-L., Wan S.-R., An Y., Wu Q., Xing Y.-H., Deng C.-H., Zhang P.-P., Long Y., Xu B.-T., Jiang Z.-Z. (2024). Targeting cell death in NAFLD: Mechanisms
and targeted
therapies. Cell Death Discovery.

[46] Yu Y., Yan Y., Niu F., Wang Y., Chen X., Su G., Liu Y., Zhao X., Qian L., Liu P., Xiong Y. (2021). Ferroptosis: A cell death connecting oxidative stress,
inflammation
and cardiovascular diseases. Cell Death Discovery.

[47] Xie Y., Hou W., Song X., Yu Y., Huang J., Sun X., Kang R., Tang D. (2016). Ferroptosis:
process and function. Cell Death Differ..

[48] Chen X., Li J., Kang R., Klionsky D. J., Tang D. (2021). Ferroptosis: machinery
and regulation. Autophagy.

[49] Xie Y., Kang R., Klionsky D. J., Tang D. (2023). GPX4 in cell death,
autophagy, and disease. Autophagy.

[50] Koppula P., Zhuang L., Gan B. (2021). Cystine transporter SLC7A11/xCT in
cancer: ferroptosis, nutrient dependency, and cancer therapy. Protein Cell.

[51] Lu S., Liu Z., Qi M., Wang Y., Chang L., Bai X., Jiao Y., Chen X., Zhen J. (2024). Ferroptosis and its
role in osteoarthritis: Mechanisms, biomarkers, and therapeutic perspectives. Front. Cell Dev. Biol..

[52] Dodson M., Castro-Portuguez R., Zhang D. D. (2019). NRF2 plays a critical role in mitigating
lipid peroxidation and ferroptosis. Redox Biol..

[53] Hanna V. S., Hafez E. A. A. (2018). Synopsis of arachidonic
acid metabolism: A review. J. Adv. Res..

[54] Zhang B. B., Zhou G., Li C. (2009). AMPK: an emerging
drug target for
diabetes and the metabolic syndrome. Cell Metab..

[55] Feng J., MengHuan L., TingTing Y., XueJie Y., HaiNing G. (2025). Research progress
on AMPK in the pathogenesis and treatment of MASLD. Front. Immunol..

[56] Han Y., Wang X., Yu D. (2025). Roflumilast
inhibits neuronal ferroptosis
via AMPK/Nrf2/HO-1 signaling and promotes motor function recovery
after spinal cord injury in rats. Cell. Signalling.

[57] Wang X., Chen X., Zhou W., Men H., Bao T., Sun Y., Wang Q., Tan Y., Keller B. B., Tong Q. (2022). Ferroptosis is essential
for diabetic cardiomyopathy and is prevented
by sulforaphane via AMPK/NRF2 pathways. Acta
Pharm. Sin. B.

[58] Kim H. S., Kim M. J., Kim E. J., Yang Y., Lee M. S., Lim J. S. (2012). Berberine-induced AMPK activation
inhibits the metastatic
potential of melanoma cells via reduction of ERK activity and COX-2
protein expression. Biochem. Pharmacol..

[59] Hwang J. T., Ha J., Park O. J. (2005). Combination of 5-fluorouracil and genistein induces
apoptosis synergistically in chemo-resistant cancer cells through
the modulation of AMPK and COX-2 signaling pathways. Biochem. Biophys. Res. Commun..

[60] Im J. Y., Kim D., Paik S. G., Han P. L. (2006). Cyclooxygenase-2-dependent neuronal
death proceeds via superoxide anion generation. Free Rad. Biol. Med..

[61] Garcia D., Shaw R. J. (2017). AMPK: Mechanisms
of Cellular Energy Sensing and Restoration
of Metabolic Balance. Mol. Cell.

[62] Shao D., Oka S., Liu T., Zhai P., Ago T., Sciarretta S., Li H., Sadoshima J. (2014). A redox-dependent mechanism for regulation of AMPK
activation by Thioredoxin1 during energy starvation. Cell Metab..

