# Supplementary material for: Oleanolic Acid Ameliorates Metabolic Dysfunction-Associated Steatotic Liver Disease by Inhibiting Ferroptosis through Targeting PTGS2 as a Key Molecular Node and Activating the AMPK/ACC Signaling Pathway
Source: J Agric Food Chem. 2026 Jul 1;74(27):21141–58. doi: 10.1021/acs.jafc.5c17633 (PMC13383740; doi:10.1021/acs.jafc.5c17633)
Supplement: Supplementary file 1 [file jf5c17633_si_001.pdf]

Supporting Information.

Oleanolic Acid Ameliorates Metabolic Dysfunction-associated Steatotic Liver Disease by Inhibiting Ferroptosis through Targeting PTGS2 as a Key Molecular Node and Activating the AMPK/ACC Signaling Pathway

Wei Ye[1#], Lei Wang[1,2#], Ye Fan[1], Li Ma[1], Zaohong Chen[1], Jinmeng Zhang[3,4], Rong Zeng[1,2], Xiang Liu[1,2*]

[1]Department of Laboratory Medicine, Hubei University of Chinese Medicine, Wuhan, Hubei 430065, China

[2]Hubei Shizhen Laboratory, Hubei University of Chinese Medicine, Wuhan, Hubei 430065, China

[3]Department of Chinese Medicine, Hubei University of Chinese Medicine, Wuhan, Hubei 430065, China

[4]Department of Spleen and Gastroenterology, Hubei Provincial Hospital of Traditional Chinese Medicine, Hubei University of Chinese Medicine, Wuhan, Hubei 430061, China

[#]Co-first author [*]Co-corresponding author

[*]Correspondence to：Dr Xiang Liu, Department of Laboratory Medicine, Hubei University of Chinese Medicine, 16 Huangjiahu West Road, Wuhan, Hubei 430065, P.R. China

E-mail: 1568@hbucm.edu.cn

Table S1 Ingredient List of 60 kcal% Fat High-Fat Diet (HFD) for Mice

Ingredient	Energy Density (kcal/g)	Weight (gram)	Total Energy (kcal)
Casein	4	200	800
L-Cystine	4	3	12
Sucrose	4	68.8	275
Maltodextrin	4	125	500
Lard	9	245	2205
Soybean Oil (contains TBHQ)	9	25	225
Cellulose	0	50	0
Mineral Mix #210088	1.6	10	16
Calcium Carbonate	0	5.5	0
Dicalcium Phosphate	0	13	0
Potassium Citrate H2O	0	16.5	0
Vitamin Mix #300050	3.9	10	39
Choline Bitartrate	0	2	0
Blue Dye	0	0.05	0
Total		773.85	4072

Table S2. URLs of databases and bioinformatics websites used in this study

name	website address
PubChem database	https://pubchem.ncbi.nlm.nih.gov/
SwissTargetPrediction database	http://swisstargetprediction.ch/
bioinformatics website	http://www.bioinformatics.com.cn
STRING database	https://string-db.org/
MicroBioinformatics website	http://www.bioinformatics.com.cn
RCSB PDB database	http://www.rcsb.org/

Table S3 Sequences for SQ-PCR primers

Primers	Forward (5'-3')	Reverse (5'-3')	Length(bp)
GPX4(human)	GAAGCAGGAGCCAGGGAGT	ACGCAGCCGTTCTTGTCG	210
SLC7A11(human)	TGCTGGGCTGATTTTTCTCCG	GAAAGGGCAACCATGAAGAGG	114
Nrf2(human)	CACATCCAGTCAGAAACCAGTGG	GGAATGTCTGCGCCAAAAGCTG	112
TFR1(human)	GCTTTCCCTTTCCTTGCA	CGAACTGACCAGCGACCT	172
AMPK (human)	TCCGAGGAAATCAAGGCACC	GCCAAGCTGGCTGGTTACTA	297
ACC (human)	TGGTAATGCGGTATGGAAGTCG	TGTATGTTGTCCCTAAGGATTGTGC	309
PTGS2(human)	TGAATGGGGTGATGAGCAGT	GGGATGCCAGTGATAGAGGG	205
GAPDH (human)	GTCTCCTCTGACTTCAACAGCG	ACCACCCTGTTGCTGTAGCCAA	131

Table S4. Antibodies and reagents used for Western blot analysis

Name	Catalog No.	Dilution	Clone No.	Manufacturer
10% SDS-PAGE gel	G2043,			Servicebio
PVDF membrane	IPVH00010			EMD Millipore
skim milk powder	GC310001			Servicebio
anti-GAPDH rabbit monoclonal antibody	AB181602	1:10,000	EPR16891	ABCAM
anti-PTGS2 polyclonal antibody	WL01750	1:1000		Wanlei Life Sciences
anti-AMPK-α rabbit polyclonal antibody	AF6423	1:1000		Affinity Biosciences
Anti-phospho-AMPK-α(Thr172) rabbit polyclonal antibody	AF3423	1:1000		Affinity Biosciences
Anti-ACC1 rabbit monoclonal antibody	ET1609-77	1:1000	ST53-08	HuaAn Biotechnology
Anti-phospho-ACC1 (S79) rabbit monoclonal antibody	HA721714	1:1000	JE63-95	HuaAn Biotechnology
Anti-GPX4 rabbit monoclonal antibody	T56959	1:1000		Abmart
Anti-SLC7A11 rabbit monoclonal antibody	T57046	1:1000		Abmart
Anti-Nrf2 polyclonal antibody	WL02135	1:1000		Wanlei Life Sciences
Anti-TFR1 rabbit monoclonal antibody	R381603	1:1000	R08-9V6	Zen Bioscience
